# Aging Affects Pancreatic α‐Cell Function and Promotes Hyperglucagonemia: Implications in Age‐Associated Diabetes

**DOI:** 10.1111/acel.70753

**Published:** 2026-10-06

**Authors:** Eva Tudurí, Lucía Almagro, Ana Ojeda‐Rodríguez, Raquel Pascua‐Maestro, Henver S. Brunetta, Sergi Soriano, Alejandro López‐Moreno, Talía Boronat‐Belda, Sergio Velasco‐Avilés, Joel Alves da Silva Junior, Manuel Castellano‐Muñoz, Alex Rafacho, Irene Cózar‐Castellano, Ángel Nadal, Paloma Alonso‐Magdalena, Beatriz Merino, José López‐Miranda, Ivan Quesada

**Affiliations:** ^1^ Instituto de Investigación, Desarrollo e Innovación en Biotecnología Sanitaria de Elche (IDiBE) Universidad Miguel Hernández de Elche Elche Spain; ^2^ CIBER de Diabetes y Enfermedades Metabólicas Asociadas (CIBERDEM) Instituto de Salud Carlos III Madrid Spain; ^3^ Departamento de Fisiología, Genética y Microbiología Universidad de Alicante Alicante Spain; ^4^ Lipids and Atherosclerosis Unit, Internal Medicine Unit Reina Sofia University Hospital Cordoba Spain; ^5^ Maimonides Biomedical Research Institute of Cordoba (IMIBIC) Cordoba Spain; ^6^ CIBER Fisiopatología de la Obesidad y Nutrición (CIBEROBN) Instituto de Salud Carlos III Madrid Spain; ^7^ Unidad de Excelencia Instituto de Biomedicina y Genética Molecular (IBGM) Consejo Superior de Investigaciones Científicas (CSIC) y Universidad de Valladolid (UVa) Valladolid Spain; ^8^ Department of Molecular Biosciences, the Wenner‐Gren Institute Stockholm University Stockholm Sweden; ^9^ Department of Physiological Sciences, Center of Biological Sciences Federal University of Santa Catarina Florianopolis Brazil; ^10^ Instituto de Investigación Biosanitaria de Valladolid (IBioVALL) Valladolid Spain; ^11^ Departamento de Biología Celular, Genética, Histología y Farmacología Universidad de Valladolid Valladolid Spain

**Keywords:** endocrine pancreas, glucagon release, glucose homeostasis

## Abstract

Aging is a major risk factor in the development of type 2 diabetes mellitus (T2DM). With age, pancreatic β‐cells undergo functional and cell identity changes that can compromise insulin secretion, thereby contributing to impaired glucose tolerance and T2DM. However, glucose homeostasis also depends on pancreatic α‐cells. Excessive plasma glucagon levels, along with impaired regulation of α‐cell secretion, are involved in the pathophysiology of T2DM. Despite their importance, the effects of aging on α‐cells have been overlooked. In the present study, we analyzed pancreatic α‐cells in aged mice with normal insulin sensitivity and in those with peripheral insulin resistance. Regardless of insulin sensitivity, aged mice exhibited hyperglucagonemia and increased glucagon release. Ex vivo, these alterations were associated with increased α‐cell mass, higher glucagon content, impaired suppression of glucagon secretion, and endoplasmic reticulum stress. Furthermore, aged mice showed remodeling of islet cellular composition and changes in the spatial organization of α‐cells within the islet. Additionally, moderate alterations in α‐cell identity were noted. While most of these alterations were already present in aged mice with normal insulin sensitivity, they were generally more pronounced with insulin resistance. Examination of the CORDIOPREV clinical study revealed the presence of hyperglucagonemia in older adults with insulin resistance. Additionally, an increased risk of developing T2DM was also found in older people with higher plasma glucagon levels. The findings described here indicate that aging affects the biology of α‐cells and glucagon, positioning them as active players in impaired glucose tolerance and diabetes associated with aging.

## Graphical abstract

Aging induces hyperglucagonemia through direct effects on pancreatic α‐cells or indirect systemic effects. This correlates with increased α‐cell mass as well as various alterations in α‐cell identity and function, which are exacerbated in the context of insulin resistance.

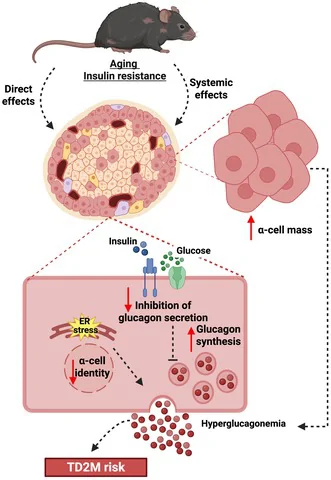

## 1 Introduction

Aging is a multifaceted process that profoundly affects metabolism, including glucose homeostasis. Along with obesity, aging constitutes one of the most critical risk factors for the development of impaired glucose tolerance (IGT) and type 2 diabetes mellitus (T2DM) (Chia et al. 2018). According to the International Diabetes Federation, the prevalence of T2DM in 2024 was 11.1% of the global population aged 20–74 years old (IDF 2025). This figure exceeds 20% in people aged 65–99 years, and projections indicate that this prevalence will continue to increase in the coming decades, highlighting the importance of aging in this disease. Insulin resistance (IR) is a key contributor to the development of T2DM (Tudurí, Soriano, Almagro, Montanya, et al. 2022). Insulin sensitivity can fluctuate throughout life, decreasing in physiological and pathological conditions such as pregnancy, obesity, and aging (Tudurí, Soriano, Almagro, Montanya, et al. 2022). In these scenarios, both pancreatic β‐cell mass and function must adapt to the body's increased insulin requirements to maintain euglycemia. Insufficient adaptation to IR plays a central role in the progression from normal glucose tolerance to IGT and, subsequently, to T2DM (Tudurí, Soriano, Almagro, Montanya, et al. 2022; Kawamori 2017). Given that aging affects peripheral insulin sensitivity (Chia et al. 2018; Tudurí, Soriano, Almagro, Montanya, et al. 2022), which in turn influences the β‐cell response, it is important to distinguish the direct effects of aging on insulin‐secreting cells from those resulting from age‐dependent IR. In this context, we have previously reported several alterations in pancreatic β‐cells from aged mice (Tudurí, Soriano, Almagro, Garcia‐Heredia, et al. 2022). Notably, most of these changes were already evident in aged animals with preserved insulin sensitivity and became more pronounced in those with IR (Tudurí, Soriano, Almagro, Garcia‐Heredia, et al. 2022). Together, these findings indicated that aging directly affects β‐cell function.

Several studies in mice and humans have specifically addressed age‐related changes in different key aspects of the pancreatic β‐cell biology. These alterations involve, among others, proliferation, apoptosis, glucose metabolism, electrical activity, and Ca^2+^ signaling, thereby affecting β‐cell survival and insulin secretion (Park et al. 2025; Tudurí, Soriano, Almagro, Garcia‐Heredia, et al. 2022). Recent findings in mice and humans have also demonstrated that compromised β‐cell function during aging is associated with endoplasmic reticulum (ER) stress and changes in critical β‐cell transcription factors that regulate β‐cell identity and maturation (Shrestha et al. 2022; Aguayo‐Mazzucato et al. 2017; Enge et al. 2017; Avrahami et al. 2015; Brusco et al. 2023). These alterations are thought to restrain the adaptive response of β‐cells to cellular stress and age‐related changes in insulin sensitivity, promoting impaired glucose homeostasis.

Most studies on the regulation of glucose metabolism in aging have focused on β‐cells and insulin actions. However, pancreatic α‐cells and glucagon secretion are also key to glucose homeostasis and metabolism. In response to hypoglycemia, α‐cells increase glucagon release to restore glucose levels by modulating hepatic glucose production; in contrast, hyperglycemic conditions inhibit α‐cell secretion (Gilon 2020; Gromada et al. 2018). Furthermore, intra‐islet glucagon signaling plays an important function in the modulation of insulin secretion (Svendsen et al. 2018). Of note, α‐cell dysfunction is implicated in the pathophysiology of T2DM (Gilon 2020; Gromada et al. 2018; Capozzi et al. 2022). Absolute or relative basal hyperglucagonemia as well as impaired α‐cell inhibition by glucose have been reported in T2DM patients, contributing to excessive hepatic signaling and hyperglycemia (Gilon 2020; Gromada et al. 2018; Capozzi et al. 2022). In this context, pharmacological modulation of glucagon signaling has attracted considerable interest as a potential therapeutic approach in T2DM (Capozzi et al. 2022).

Despite the importance of glucagon and pancreatic α‐cells in glucose homeostasis and T2DM, the effects of aging on these islet cells remain unexplored. In the present study, we have examined pancreatic α‐cells in aged mice with normal insulin sensitivity and in those with IR. Hyperglucagonemia was observed in aged mice, which was accompanied by increased α‐cell mass, excessive glucagon release, and impaired inhibition of α‐cell secretion, along with endoplasmic reticulum (ER) stress. Furthermore, alterations in α‐cell composition and spatial organization within the islets were identified. Most of these effects were more pronounced in insulin‐resistant aged mice. Of note, in the CORDIOPREV clinical study, we found that hyperglucagonemia was associated with aging in individuals with IR. Additionally, the highest risk of developing diabetes over time was found in older adults with higher plasma glucagon levels. Overall, these findings indicate that aging disrupts α‐cell biology and plasma glucagon levels, which may contribute to metabolic dysregulation associated with advancing age.

## 2 Material and Methods

### 2.1 Experimental Animals

Young adult (3‐month‐old ±2 weeks) and aged (20‐month‐old ±2 weeks) male mice (C57BL/6JOlaHsd, Envigo, Barcelona, Spain) were housed under standardized environmental conditions (12‐h light/dark cycle, 22°C, unrestricted access to food and water). All experimental procedures were reviewed and approved by the Animal Ethics Committee of Miguel Hernández University and by the competent regional authority (ref. 2016/VSC/PEA/00116). Experiments were carried out in compliance with current European legislation. The animals were sourced from the institution's animal facility.

### 2.2 QUICKI Index and Plasma Measurements of Glucose, Insulin, and Glucagon

QUICKI index was calculated as 1/[log (G0) + log (I0)], where G0 (mg/dL) and I0 (μU/mL) represent 6 h fasting blood glucose and plasma insulin, respectively. Aged mice were divided into two groups: high insulin sensitive (HIS) or low insulin sensitive (LIS), according to whether their individual QUICKI index fell over (LIS) or below (HIS) the 25th percentile of the corresponding averaged QUICKI value of the control group, respectively (Tudurí, Soriano, Almagro, Garcia‐Heredia, et al. 2022; Hauth et al. 2011). We have previously described the stratification and characterization of these aged groups in relation to glucose homeostasis and insulin levels (Tudurí, Soriano, Almagro, Garcia‐Heredia, et al. 2022). Fasting plasma glucagon and insulin were determined from blood extracted from the saphenous vein and using a glucagon mouse ELISA (Crystal Chem Inc., Cat#81518, Elk Grove Village, IL, USA) and an insulin mouse ultrasensitive ELISA (Crystal Chem Inc. Cat.#90080, Elk Grove Village, IL, USA). Blood glucose levels were monitored using an automatic glucometer (Accu‐Chek Compact plus, Roche, Madrid, Spain). For the arginine tolerance test, 6 h‐fasted mice were intraperitoneally injected with L‐arginine (2 g/kg body weight).

### 2.3 Islet Isolation and Cell Culture

Mice were sacrificed via cervical dislocation, followed by islet isolation using collagenase digestion (Tudurí, Soriano, Almagro, Garcia‐Heredia, et al. 2022). For certain experiments, isolated islets were further dissociated into single cells using trypsin digestion and then cultured overnight at 37°C in RPMI 1640 medium (Gibco, Thermo Fisher Scientific Inc., Carlsbad, CA) supplemented with 10% fetal calf serum, 100 IU/mL penicillin, 0.1 mg/mL streptomycin, and 11 mM D‐glucose (Tudurí, Soriano, Almagro, Garcia‐Heredia, et al. 2022).

### 2.4 Ex Vivo Glucagon Secretion and Glucagon Content Measurements

Freshly isolated islets were allowed to recover at 37°C for 2 h (Junior et al. 2025). Subsequently, groups of 15 size‐matched islets were placed in a 24‐well plate with Millicell Cell Culture Inserts (Merk, Darmstadt, Germany) immersed in 500 μL of a solution containing (in mM): 140 NaCl, 4.5 KCl, 2.5 CaCl_2_, 1 MgCl_2_, 20 HEPES, D‐glucose (0.5 or 11 mM) and/or insulin (10 nM), pH = 7.35, and incubated at 37°C with 5% CO_2_ for 1 h to allow glucagon secretion. The medium was then collected and frozen for subsequent glucagon measurement by ELISA (Mercodia AB, Cat# 10‐1271‐01, Uppsala, Sweden). As in previous secretion studies, we used 0.5 or 11 mM glucose, given that low concentrations (0–1 mM) lead to maximal to submaximal ex vivo glucagon release, while intermediate levels produce a maximal to submaximal suppression (Akiyama et al. 2013; Merino et al. 2022; Junior et al. 2025). Islets were then handpicked and transferred to 20 μL of ethanol/HCl buffer, followed by overnight incubation at 4°C for quantification of glucagon content. Protein concentration was determined using the Bradford dye method and used to normalize both glucagon secretion and glucagon content (Junior et al. 2025).

### 2.5 Electrophysiological Recordings

Voltage‐gated K^+^ and Ca^2+^ currents were simultaneously recorded using standard patch‐clamp recordings procedures from isolated α‐cells in whole cell configuration. Pancreatic α‐cells were identified by size (< 5 pF) and the corresponding steady‐state inactivation properties of the tetrodotoxin‐sensitive Na^+^ current (Junior et al. 2025). Data were obtained using Axopatch 200B amplifier (Axon Instruments Co. Union City, CA, USA). The bath solution contained (in mM): 5 KCl, 135 NaCl, 2.5 CaCl_2_, 10 HEPES, and 1.1MgCl_2_ and was supplemented with 5 mM Glucose (pH 7.4 with NaOH). The pipette was filled with (in mM) 120 KCl, 1 MgCl_2_, 1CaCl_2_, 3 MgATP, 10 EGTA, and 10 HEPES (pH 7.15 with KOH). K_v_ and Ca^2+^ currents were recorded in response to depolarizing voltage pulses of −60 to +80 mV from a holding potential of −70 mV. Data were filtered (2KHz) and digitalized (10KHz) using a Digidata 1550B (Molecular Devices, San Jose, CA, USA). For currents density quantification, currents (in pA) were normalized to the cell capacitance (in pF).

### 2.6 Pancreas Preparation and α‐Cell Mass Quantification

Following removal, pancreata were weighted and fixed in ice‐cold 4% paraformaldehyde for 20 h at 4°C. After fixation, the pancreata were embedded in paraffin, and 5 μm sections were cut at intervals of 200 μm. For α‐cell mass analysis, antigen retrieval was performed by heating the sections for 20 min in citrate buffer (10 mM, pH 6.0), followed by blocking in 3% BSA in PBS (2 h, RT). The sections were then incubated overnight at 4°C with a rabbit anti‐glucagon antibody (1:100, MON‐RTU1115, Monosan, Uden, The Netherlands), followed by incubation with biotinylated goat anti‐rabbit IgG (H + L) (Zymed, BA 9200, Vector Laboratories, Burlingame, CA, USA). The α‐cell area was determined by measuring the total glucagon‐stained area and normalizing it to the total pancreatic area using Metamorph Software (Molecular Devices, San Jose, CA, USA). Finally, α‐cell mass was calculated by multiplying the α‐cell area (%) by the total pancreas weight.

### 2.7 Apoptosis and α‐Cell Proliferation Analysis

Apoptotic α‐cells were identified using the TUNEL technique (In Situ Cell Death Detection Kit, Roche, Switzerland), after overnight incubation at 4°C with a mouse anti‐glucagon antibody (1:1000, G2654, Sigma‐Aldrich, Madrid, Spain), followed by incubation (2 h, RT) with Alexa Fluor 546 (1:500, Life Technologies, Carlsbad, CA, USA). Proliferation was assessed by overnight incubation with a mouse anti‐glucagon antibody (1:1000, G2654, Sigma‐Aldrich, Madrid, Spain) and a rabbit anti‐Ki67 antibody (1:400, 12202S, Cell Signaling Technology, Danvers, MS, USA), followed by incubation (2 h, RT) with Alexa Fluor 546 and 488 (1:500, Life Technologies, USA). Nuclei were counterstained with Hoechst 33342 (1:1000; Invitrogen, Barcelona, Spain). Images were captured using an IN Cell Analyzer 6000 system (GE Healthcare, Little Chalfont, UK), and data analysis was performed using ImageJ software (National Institutes of Health, Bethesda, MD, USA).

### 2.8 Immunofluorescence Analysis

Paraffin pancreas sections were dewaxed in xylene, rehydrated through ethanol series and washed in PBS. For hormone immunofluorescence analysis, antigen retrieval was performed in a pressure cooker for 30 min in Citrate buffer solution (pH = 6), followed by blocking (1 h, RT) in 10% fatty acid free‐BSA PBS (blocking solution) supplemented with 1% (v/v) normal goat serum and 0.05% (v/v) TRITON‐100X. Pancreas slides were incubated overnight at 4°C with mouse monoclonal anti‐insulin (Sigma Ref: I2018; 1:1000) and rabbit monoclonal anti‐glucagon (Abcam Ref: AB92517; 1:2000) in blocking solution. For Arx, Pdx‐1, Xbp1 and BiP, antigen retrieval was performed by boiling slides in Tris–EDTA buffer (1:10 dilution; pH 9.0) for 40 min, followed by blocking (1 h, RT) in PBST (PBS containing 0.2% Tween‐20) supplemented with 3% FBS (FBS blocking solution). Pancreas sections were incubated overnight at 4°C with rabbit anti‐ARX (1:200, ab308260, Abcam, Cambridge, UK), guinea pig anti‐PDX‐1 (1:250, ab47308, Abcam, Cambridge, UK), rabbit anti‐XBP1 (1:100, AB37152, Abcam, Cambridge, UK) or rabbit anti‐BiP (1:100, AB21685, Abcam, Cambridge, UK), and mouse anti‐glucagon (1:2000, ab10988, Abcam, Cambridge, UK) in FBS blocking solution. Alexa Fluor 594, or 488‐conjugated IgGs (Invitrogen) were used as secondary antibodies (1:1000). Preparations were mounted with Fluoroshield with Dapi (F6057, Sigma‐Aldrich, Madrid, Spain). Confocal images were acquired using a 40× oil immersion objective on a confocal DMI 6000B microscope equipped with a TCS SP5 confocal system (Leica) or, in the case of Xbp1 and BiP, a ZEISS LSM 900 with Airyscan 2. Images were processed and analyzed using FIJI software. To analyze XBP1 and BiP fluorescence intensity, we opened the image channels separately and generated a binary mask by adjusting the threshold on the glucagon channel to define the region of interest (ROI). Then, we superimposed this mask onto the XBP1 or BiP channels to accurately measure their signal within the α‐cells.

### 2.9 Transmission Electron Microscopy (TEM)

Freshly isolated pancreatic islets were prepared using the standard Spurr embedding protocol (Tudurí, Soriano, Almagro, Garcia‐Heredia, et al. 2022). Thin sections were cut using an ultramicrotome (Leica UC7; Leica Microsystems, Germany), and TEM imaging was performed with an EMCCD camera (TRS 2 k × 2 k) on a Zeiss Libra 120 TEM operating at 80 kV, as previously reported (Tudurí, Soriano, Almagro, Garcia‐Heredia, et al. 2022). A minimum of 10 TEM images from both control and aged islets (*n* = 3 mice/group) were acquired and analyzed with ImageJ software (National Institutes of Health, Bethesda, MD, USA). Pancreatic α‐cells were easily identified by their characteristic ultrastructural features and typical secretory granules, as previously described (Tudurí, Soriano, Almagro, Garcia‐Heredia, et al. 2022; Marroqui et al. 2015; Masini et al. 2017).

### 2.10 RNA Isolation and Gene Expression Analysis by RT‐qPCR

Total RNA was isolated using the RNeasy Micro Kit (Qiagen, Madrid, Spain) according to the manufacturer's protocol and quantified using a Nanodrop 2000 spectrophotometer (Thermo Fisher Scientific, Waltham, MA, USA). cDNA synthesis was performed using the High Capacity cDNA Reverse Transcription Kit (Applied Biosystems, Foster City, CA, USA). Quantitative RT‐PCR (qRT‐PCR) was carried out in a final volume of 10 μL, comprising 1 μL of cDNA, 200 nM of each primer, and 1 x IQ SYBR Green Supermix (Bio‐Rad, Hercules, CA, USA), using a CFX96 Real‐Time System (Bio‐Rad, Hercules, CA, USA). Relative gene expression levels were determined using the 2^−ΔΔCt^ method. Primer sequences are listed in Table S1.

### 2.11 Analysis of Single‐Cell Transcriptomics Data

Single‐cell analysis was performed using a publicly available dataset (Hrovatin et al. 2023). Seurat v5 files were downloaded, and cell identities and annotations were retained as defined by the original authors. Samples were stratified into two age groups. The young group comprised mice aged 5 weeks, 8 weeks, 2–3 months, and 4 months. The old group consisted of 2‐year‐old mice. After subgroup extraction, ER stress–related genes and cell identity markers were retrieved from pancreatic α‐cells as previously identified in the original study. Comparisons between groups were performed using a two‐tailed Student's *t*‐test, followed by false discovery rate correction. Single‐cell RNA‐seq data were reanalyzed in Python using a Jupyter Notebook–based workflow. Codes used to reanalyze single‐cell data are available at: github.com/henver‐brunetta/alphacells_aging.

## 3 CORDIOPREV Study

### 3.1 Population

The current work was conducted within the framework of the CORDIOPREV study (Clinicaltrials.gov NTC00924937). All the patients gave written informed consent to participate in the trial. The protocol and all amendments were approved by the local ethics committees, following the Helsinki declaration and good clinical practices (Roncero‐Ramos et al. 2018; Pozuelo‐Sanchez et al. 2020). In this work, we included 462 of 1002 patients from the CORDIOPREV study who were not diagnosed with T2DM at baseline. Of these, 107 patients developed T2DM (Incident‐T2DM) according to the American Diabetes Association (ADA) diagnostic criteria (ADA 2014) after a median follow‐up of 60 months. Nineteen patients were excluded from analyses involving telomere length (TL) due to missing data, resulting in a final sample of 338 non‐T2DM and 105 Incident‐T2DM patients. T2DM incidence was assessed annually using fasting plasma glucose ≥ 126 mg/dL, 2‐h plasma glucose ≥ 200 mg/dL during a 75 g OGTT, or HbA1c ≥ 6.5%.

### 3.2 Assessment of Glycemic and Metabolic Parameters

After a 12‐h overnight fast, venous blood samples were collected into EDTA tubes (final concentration EDTA, 0.1%). Plasma was separated from the red cells by centrifugation at 1500 × g for 15 min at 4°C and immediately frozen at −80°C until analysis. Biochemical determinations were performed at the Reina Sofia University Hospital by personnel blinded to the intervention. Fasting glucose was measured by the hexokinase method using spectrophotometric detection in an Architect c‐16000 analyzer (Abbott, Chicago, IL, USA). Fasting plasma insulin concentrations were determined by microparticle enzyme immunoassay (Abbott Diagnostics, Matsudo‐shi, Japan). Fasting glucagon levels were measured using a sandwich ELISA according to the manufacturer's instructions. All ratios were calculated individually for each participant by dividing the corresponding variables (e.g., fasting glucagon/fasting insulin and fasting glucagon/fasting glucose), and group values are presented as the mean of these individual ratios. For AUC‐glucagon determination, a standard oral glucose tolerance test (OGTT) was performed after an overnight fast using the Matsuda and DeFronzo method (Matsuda and DeFronzo 1999). Blood samples were collected at fasting state and after 30, 60, 90, and 120 min of the glucose overload (75 g oral glucose). Insulin resistance and insulin sensitivity were estimated using the Homeostasis Model Assessment of Insulin Resistance (HOMA‐IR = fasting insulin×fasting glucose/405) (Song et al. 2007).

### 3.3 DNA Isolation and Telomere Length Measurement

Telomere length (TL) has been previously validated in the CORDIOPREV study as a biomarker of biological aging and has consistently predicted clinically relevant outcomes, including incident T2DM, diabetes remission, and subclinical atherosclerosis (Ojeda‐Rodriguez et al. 2023, 2024). Genomic DNA was isolated from the buffy coat fraction using the salting‐out method (Miller et al. 1988), and TL was determined by quantitative PCR as described by Cawthon (2002). Detailed protocols for both procedures have been previously described and validated in earlier publications (Ojeda‐Rodriguez et al. 2024). Patients were classified by biological age based on the 20th percentile of TL, as previously validated in the CORDIOPREV study and consistently applied in our earlier studies (Ojeda‐Rodriguez et al. 2023, 2024). Individuals with TL below this cutoff were considered at risk of short telomeres, as previously defined in earlier studies (Ojeda‐Rodriguez et al. 2023), whereas those at or above the 20th percentile were considered not at risk.

### 3.4 Data Analysis in the CORDIOPREV Study

Further information can be found elsewhere (Roncero‐Ramos et al. 2018; Pozuelo‐Sanchez et al. 2020). Briefly, statistical analyses were performed using ANCOVA models to compare glycemic and metabolic parameters across four groups defined by incident T2DM and two complementary age stratification approaches. Biological age was estimated by TL using the 20th percentile cutoff. Chronological age was categorized using the 25th percentile (cutoff at 51 years) to obtain groups of comparable size to those generated by the biological age classification, thereby facilitating comparisons between both approaches. Since chronological and biological age capture complementary aspects of the aging process and do not necessarily overlap, analyses were performed using both stratification approaches independently. Continuous variables are presented as mean ± standard deviation (SD), and analyses were adjusted for potential confounders, including sex, body weight, statin treatment, and family history of diabetes. Between‐group differences were assessed using post hoc pairwise comparisons with Bonferroni correction, with statistical significance set at *p* < 0.05. In addition, T2DM incidence was evaluated using Kaplan–Meier failure curves, and Cox proportional hazards regression models were applied to estimate hazard ratios (HRs) and 95% confidence intervals (CIs), adjusted for the same covariates.

### 3.5 Statistical Analysis

Normality of data was assessed prior to performing statistical tests. One‐way ANOVA or two‐way ANOVA was used as appropriate, followed by Tukey's post hoc test for normally distributed variables. For non‐normally distributed variables, the Kruskal–Wallis test was employed, followed by Dunn's multiple comparisons test. All statistical analyses were conducted using Prism software (GraphPad Software v7). Data are presented as mean ± SEM, and statistical significance was determined at *p* < 0.05.

## 4 Results

### 4.1 Aged Mice Present Hyperglucagonemia and Enhanced In Vivo α‐Cell Secretion

Insulin sensitivity in metabolic tissues affects not only β‐cells but also α‐cells (Færch et al. 2016). To distinguish the effects of aging itself from those resulting from age‐associated IR, old mice were first divided into two groups based on their insulin sensitivity, as assessed by their QUICKI index (Figure 1A), as previously described (Tudurí, Soriano, Almagro, Montanya, et al. 2022). The HOMA‐IR index further evidenced the differences in insulin sensitivity among groups (Figure 1B). Consistent with previous findings (Tudurí, Soriano, Almagro, Montanya, et al. 2022), aged low insulin‐sensitive (aged‐LIS) mice exhibited a modest increase in blood glucose but markedly higher plasma insulin levels compared to either young adult mice (control) or aged high insulin‐sensitive (aged‐HIS) animals (Figure 1C,D, respectively). Notably, both aged groups showed hyperglucagonemia (Figure 1E) and a higher glucagon‐to‐glucose ratio (Figure 1F). We also assessed the relationship between plasma glucagon levels and insulin sensitivity (QUICKI index) across all experimental groups (Figure 1G). A negative correlation was found (*R* = −0.539, *p* < 0.0001), suggesting that IR might further contribute to fasting glucagon concentrations. To assess the in vivo α‐cell function, mice were subjected to an arginine tolerance test (Kawamori et al. 2009). While no significant differences in the AUC of the glucose curves were observed (Figure 1H), both aged groups exhibited greater insulin and glucagon responses to arginine than young controls (Figure 1I,J). Overall, these findings point to a scenario of elevated basal glucagon levels along with increased in vivo α‐cell function in aging.

**FIGURE 1:**
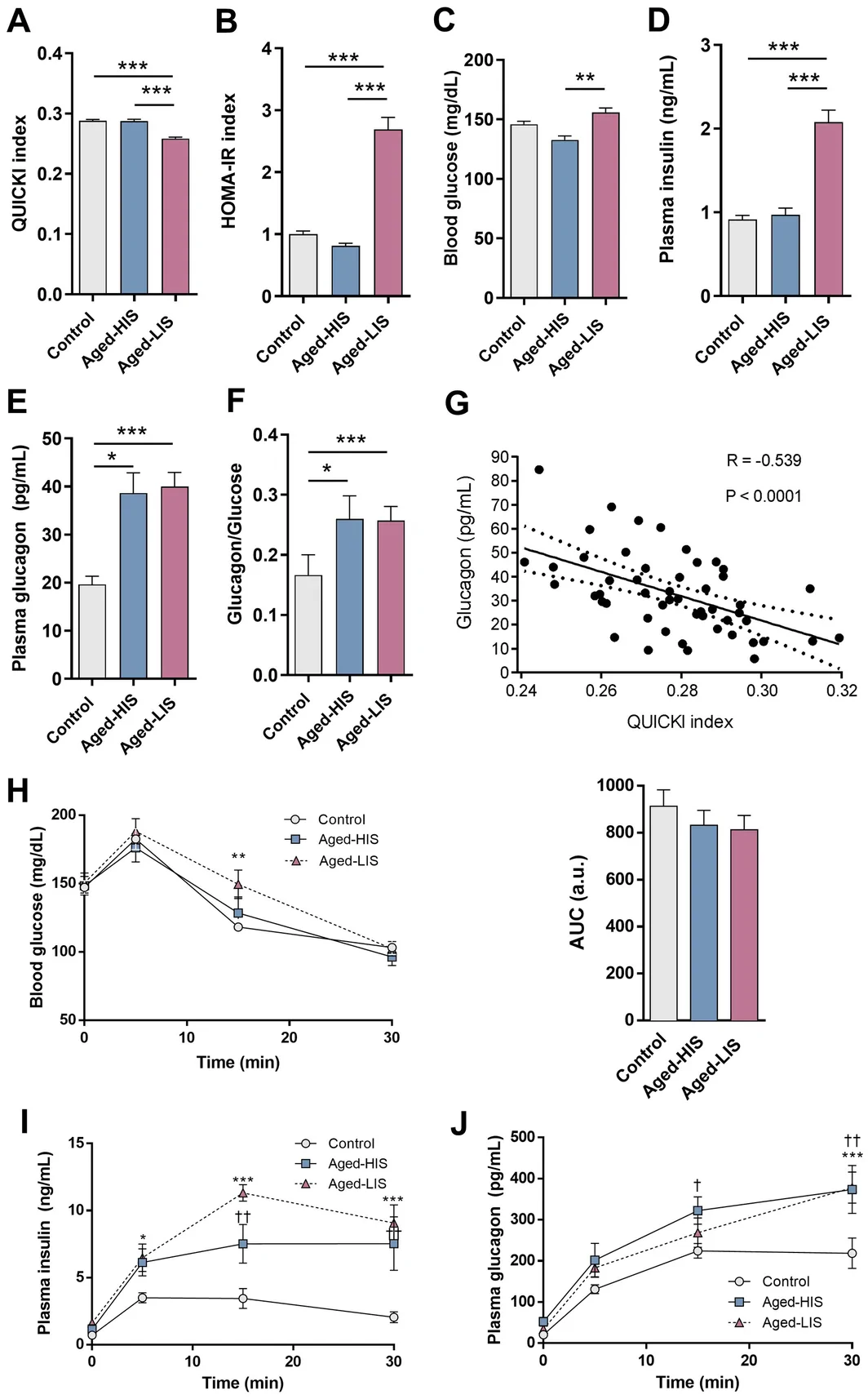
Aged mice display hyperglucagonemia and increased insulin and glucagon release in response to arginine. In vivo measurements were performed after 6 h fast. QUICKI index (A), HOMA‐IR index (B), blood glucose (C) and plasma insulin levels (D) in control (*n* = 50–54), aged‐HIS (*n* = 13), and aged‐LIS (*n* = 37–38) mice. Plasma glucagon levels (E), glucagon to glucose ratio (F) and correlation of plasma glucagon levels and QUICKI index (G) (control *n* = 24; aged‐HIS *n* = 11; and aged‐LIS *n* = 20–21 mice). (H) Blood glucose levels and AUC (control *n* = 17, aged‐HIS *n* = 7, and aged‐LIS *n* = 14), and plasma insulin (I) and glucagon (J) levels during an arginine tolerance test (control *n* = 10, aged‐HIS *n* = 5, and aged‐LIS *n* = 9). Plasma glucagon levels at *t* = 0 were higher in both aged groups compared with controls (*p* < 0.05, Kruskal–Wallis test). Data are expressed as mean ± SEM. Statistical analyses were performed using one‐way ANOVA (A, C, and E; *p < 0.05, **p < 0.01, ***p < 0.001), Kruskal‐Wallis test (B, D, F, and H [AUC]), two‐way repeated‐measures ANOVA (H, I and J; *p < 0.05, **p < 0.01, ***p < 0.001 for control vs. aged‐LIS comparisons; †*p* < 0.05, ††*p* < 0.01 for control vs. aged‐HIS comparisons), and Spearman's correlation coefficient (G).

### 4.2 Aging Affects Pancreatic α‐Cell Mass, Proliferation, and Apoptosis

Since glucagon levels are highly dependent on the α‐cell mass, we performed a morphological analysis in pancreas sections. Pancreatic α‐cell area and mass gradually increased in aged groups (*p* = 0.035 and *p* = 0.003 for significance of linear trend, respectively), with α‐cell mass being significantly higher in aged‐LIS mice (Figure 2A–C). This α‐cell mass expansion was unlikely to result from islet neogenesis or α‐cell hypertrophy, since islet density (Figure 2D) and α‐cell size (Figure 2E) remained similar across the groups. Immunofluorescence analysis of pancreas sections revealed a very low rate of apoptotic α‐cells in all groups, although it was significantly increased in aged‐LIS mice (Figure 2F,H). Aging also decreased α‐cell proliferation compared to young controls (Figure 2G,I). Overall, these findings indicate that aging results in augmented α‐cell mass (with IR exacerbating this effect), and establish a link to the hyperglucagonemia and enhanced glucagon responses observed in aged mice.

**FIGURE 2:**
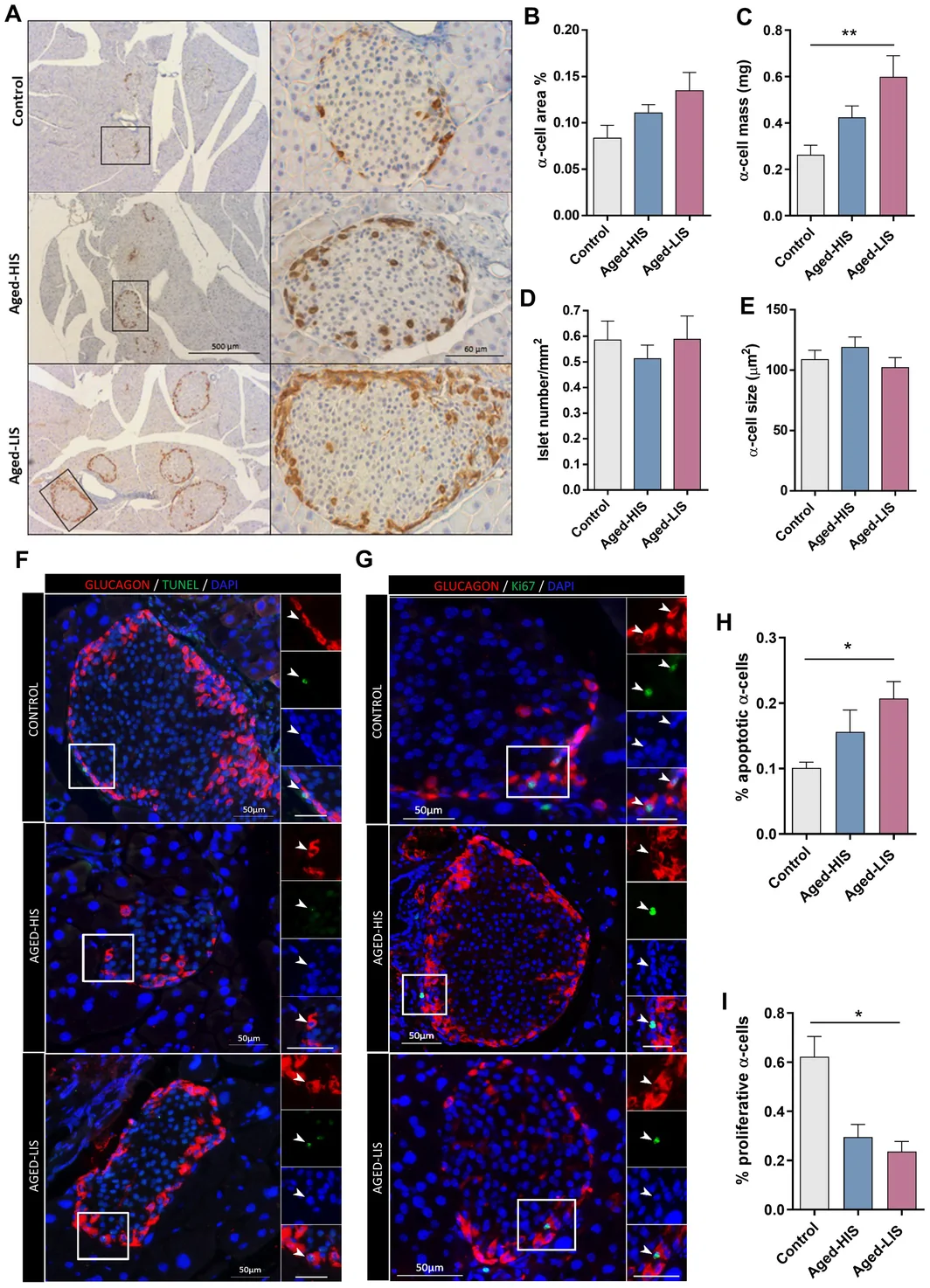
Age‐dependent morphological changes in pancreatic α‐cells. Representative images of control, aged‐HIS and aged‐LIS islets in pancreas slices (A). Pancreatic α‐cells stained with an antibody against glucagon (brown). Scale bar: 500 μm (left images), 50 μm (right images). α‐cell area (B), α‐cell mass (C), islet density (D), and α‐cell size (E) (control, *n* = 6; aged‐HIS, *n* = 5; aged‐LIS, *n* = 7). Representative images of apoptotic (F) and proliferative α‐cells (G) showing glucagon staining (red), nuclear labelling with DAPI (blue) and either proliferative apoptotic TUNEL‐positive nuclei or Ki67‐positive nuclei (green). White arrows indicate some positive nuclei. Proportion of α‐cell apoptosis (H) and proliferation (I) (*n* = 5–6 mice/group). Scale bar = 50 μm. Data are expressed as mean ± SEM. Statistical analysis were performed using one‐way ANOVA (C, D and H) and Kruskal–Wallis test (B, E, and I). *p < 0.05, **p < 0.01.

### 4.3 Aging Effects on α‐Cell Composition and Spatial Organization Within Islets

We further assessed islet cell architecture and cell type composition using immunofluorescence. Analysis of pancreas slices stained against insulin and glucagon revealed a decreased proportion of Ins^+^ cells, along with an augmented percentage of Gcg^+^ cells in the islets of aged‐LIS mice (Figure 3A–C). Moreover, the presence of Gcg^+^ cells that were not located in the characteristic islet periphery increased in aged‐LIS animals, with a marked trend observed in aged‐HIS mice (Figure 3D). Therefore, these findings point to alterations in islet cell type composition and spatial organization with aging.

**FIGURE 3:**
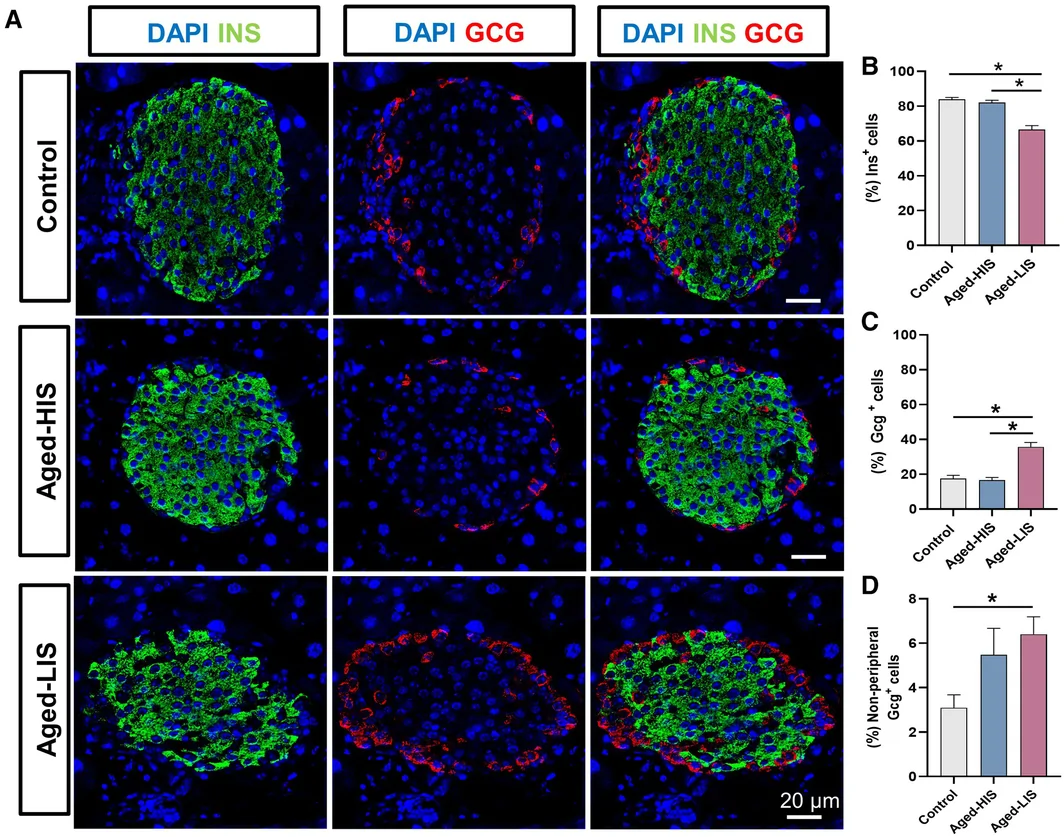
Altered α‐cell composition and spatial organization in islets from aged mice. (A) Representative immunofluorescence images of islets obtained in pancreatic sections from the three groups showing insulin (green), glucagon (red) and DAPI staining (blue). (B) Proportion of Ins^+^ cells respect to total islet cells. (C) Proportion of Gcg^+^ cells respect to total islet cells. (D) Gcg^+^ cells not located at islet periphery respect to total Gcg^+^ cells. Those Gcg^+^ cells that were not found in the outer layer of the islet or adjacent to it were counted as non‐peripheral Gcg^+^ cells (Merino et al. 2022). Scale bar = 20 μm. Data are expressed as mean ± SEM (*n* = 49–55 islets from 5 mice/group). One‐way ANOVA, *p < 0.05.

### 4.4 Aged‐LIS Mice Exhibit Impaired Inhibition of Glucagon Release by Glucose and Insulin in Pancreatic Islets

To analyze whether aging also affects α‐cell function, we assessed ex vivo glucagon secretion from freshly isolated islets under controlled conditions. Islets were exposed to low glucose (0.5 mM G), which stimulates glucagon secretion, and two inhibitory conditions: high glucose (11 mM G) and low glucose plus insulin (10 nM). As previously reported (Junior et al. 2025), control islets faced glucagon suppression under high glucose (~43%) or insulin (~54%) conditions (Figure 4A). Similar effects were observed in aged‐HIS islets (~43% for glucose and ~35% for insulin, respectively). In contrast, both stimuli failed to produce a significant (*p* > 0.05) inhibition in aged‐LIS mice (~24% and ~28%, respectively), suggesting impaired glucose metabolism and/or deficient insulin sensitivity in α‐cells. Notably, aged‐HIS islets showed higher glucagon content than controls (Figure 4B), with a similar trend in the aged‐LIS group. These changes in glucagon content do not appear to be due to abnormal preproglucagon expression (Figure 4C). We observed a pattern similar to that described in Figure 4A when normalizing secretion by glucagon content (Figure S1).

**FIGURE 4:**
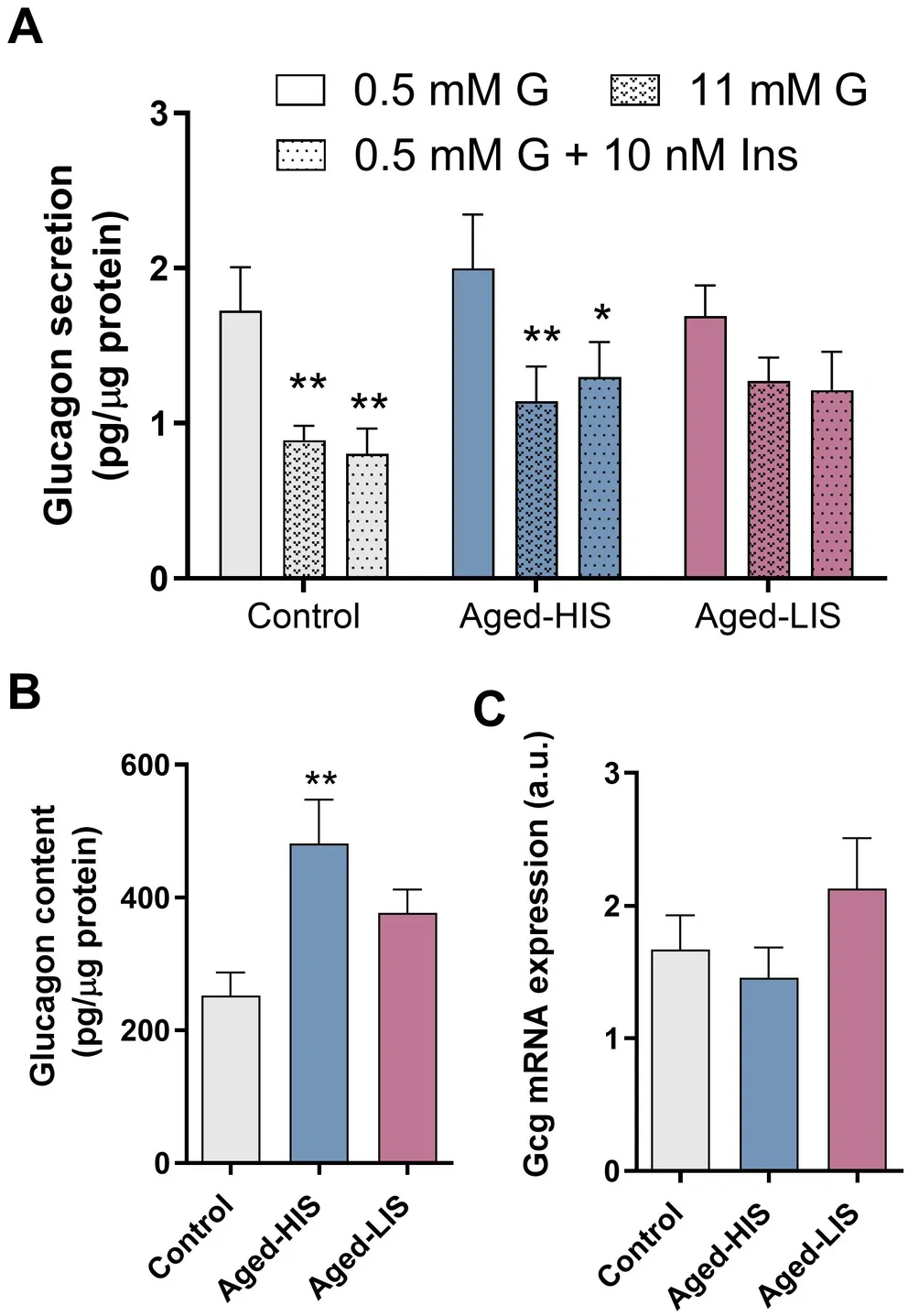
Pancreatic islets from aged‐LIS mice exhibit attenuated suppression of glucagon secretion in response to either high glucose or extracellular insulin. (A) Ex vivo static glucagon secretion from freshly isolated islets normalized by total islet protein levels (*n* = 10–11 batches from 3 mice/group). Islets were stimulated with either low glucose (0.5 mM G, stimulatory), high glucose (11 mM G, inhibitory), or low glucose and insulin (10 nM, inhibitory). (B) Glucagon protein content normalized by total islet protein levels (control, *n* = 10; aged‐HIS, *n* = 10; aged‐LIS, *n* = 12; from 3 mice/group). (C) Islet mRNA levels of the preproglucagon gene (*n* = 5 per group). Data are expressed as mean ± SEM and analyzed by two‐way ANOVA (A), one‐way ANOVA (B) and Kruskal–Wallis (C). *p < 0.05, **p < 0.01 when compared to control.

Since glucagon secretion is mediated by α‐cell electrical activity (Gilon 2020; Gromada et al. 2018), we examined whether aging affects voltage‐gated K^+^ and Ca^2+^ currents in isolated α‐cells. No differences were found among groups (Figure S2), suggesting that functional alterations in α‐cells during aging are unlikely to be associated with substantial changes in electrical activity.

To investigate whether aging alters the exocytotic machinery of α‐cells, we performed capacitance measurements using patch‐clamp. Due to model limitations, aged mice were not categorized based on insulin sensitivity in this set of experiments. In any case, α‐cells from both groups showed comparable gradual increases in capacitance in response to depolarizing pulses, with no significant differences between them (Figure S3), suggesting that the α‐cell exocytotic machinery is generally preserved in aging.

### 4.5 Ultrastructural Changes of Pancreatic α‐Cells in Aging

We examined the ultrastructure of α‐cells by TEM (Figure 5A). No significant differences among groups were observed in the number and density of glucagon granules per cell (Figure 5B,C), nor in the percentage of dense, gray and empty granules (Figure 5D), indicating that maturation and biogenesis of glucagon vesicles were generally unaffected by age. Compared with controls, aged‐LIS α‐cells displayed a modest increase in granule diameter (Figure 5E), and a reduction in the number of granules in contact with the membrane (*p* = 0.065, Figure 5F). No significant differences were found in mitochondrial area (Figure 5G). Conversely, the endoplasmic reticulum (ER) area was found to be enlarged in both aged‐HIS and aged‐LIS α‐cells compared to controls (Figure 5H). Although the organelle remained well‐structured, this finding is a representative trait of ER stress (Marroqui et al. 2015; Eizirik et al. 2020; Masini et al. 2017). To explore this further, we reanalyzed a mouse islet atlas dataset comprising over 300,000 cells from nine single‐cell RNAseq (scRNA‐seq) studies (Hrovatin et al. 2023). By pooling samples into α‐cells from young versus old mice of similar ages to those studied here, we observed increased expression of key genes involved in the unfolded protein response and in ER stress such as *Hspa5*, *Atf6*, *Ddit3*, *Xbp1*, *Ern1*, *Dnajb9*, *Herpud1*, and *Eif2ak3* in α‐cells from old mice (Figure 5I). In these scRNAseq datasets, old mice were not stratified according to IR. However, given the very similar α‐cell phenotype of aged‐HIS and aged‐LIS mice (i.e., enlarged ER size, hypersecretion, and increased glucagon content), these scRNAseq data provide single‐cell resolution level and further supports the existence of ER stress in α‐cells as a general feature of aging. Given the role of the ER proteins Xbp1 and BiP (encoded by *Hspa5*) in the α‐cell (Akiyama et al. 2013; Marroqui et al. 2015), we also analyzed their expression in pancreas sections. While no differences in Xbp1 signal intensity were found between the groups (Figure S4), BiP expression was increased in α‐cells from both aged groups compared to controls (Figure 5J,K).

**FIGURE 5:**
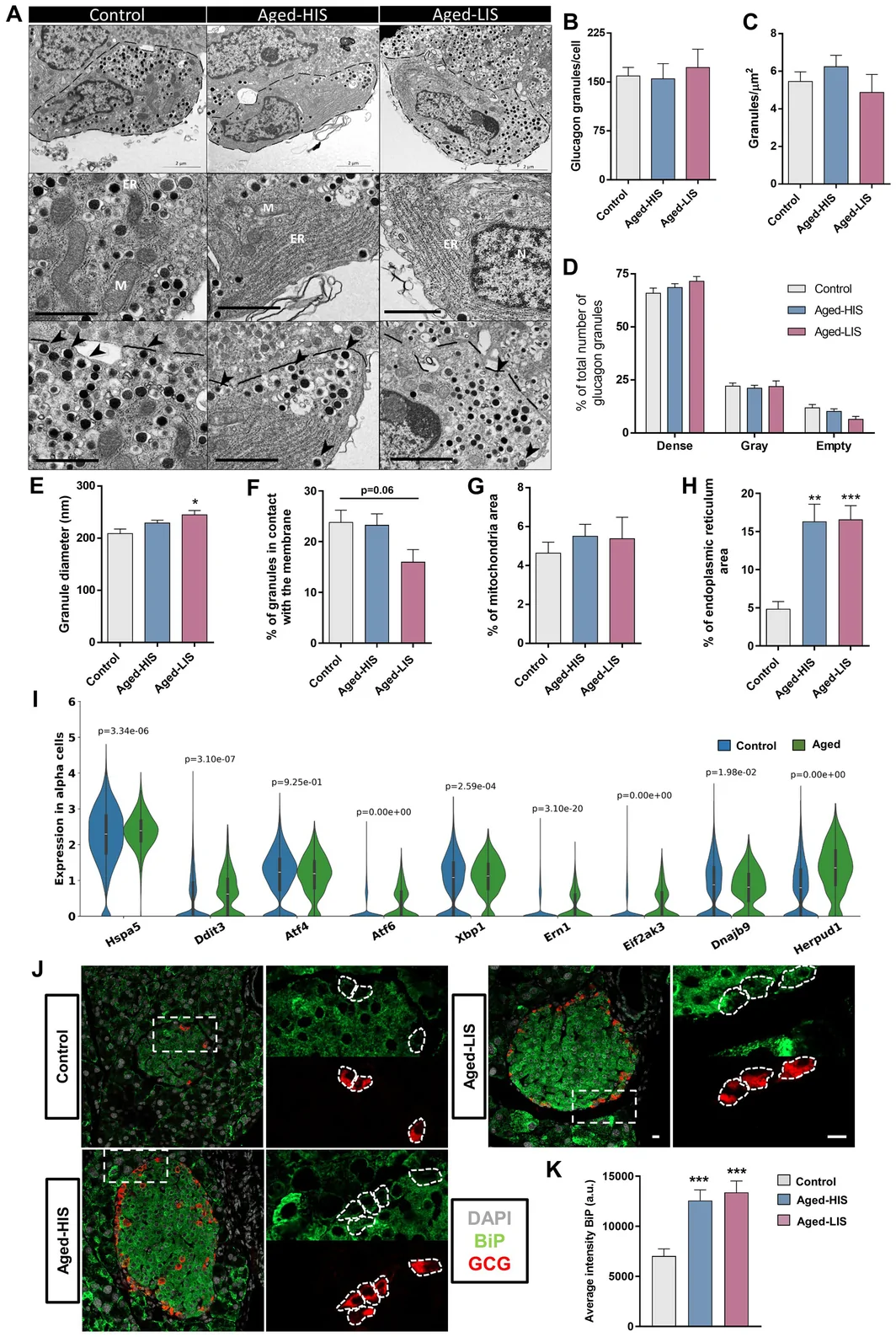
(Ultrastructure of control and aged α‐cells in electron micrographs. A) Representative electron micrographs of control, aged‐HIS, and aged‐LIS α‐cells. Top panel: Representative images of individual cells from the three groups. Middle panel: Corresponding magnified images showing some subcellular organelles. Bottom panel: Corresponding magnified images showing secretory granules. Scale bar = 2 μm (upper panel) and 1 μm (medium and lower panel). (B) Glucagon granules per cell. (C) Granule density. (D) Percentage of dense, gray and empty granules. (E) Glucagon granule diameter. (F) Percentage of glucagon granules in contact with the membrane. (G) Percentage of mitochondrial area. (H) Percentage of endoplasmic reticulum area. Control: *n* = 11–12 α‐cells; aged‐HIS: *n* = 18 α‐cells; aged‐LIS: *n* = 11 α‐cells, from 3 mice per group. (I) Analysis of scRNAseq from public datasets (Hrovatin et al. 2023) showing the expression of ER stress genes in pancreatic α‐cells from young adults and old mice. Gene expression is shown on a logarithmic scale. (J) Illustrative immunofluorescence images of islets in pancreas slices showing BiP (green), glucagon (red) and DAPI staining (gray). In each group, the white squares on the left images indicate the magnified area shown in the images on the right. The contour of α‐cells is indicated by a dashed line on the right images. Images are representative of 30–34 islets in each group from 3 different mice per group. Scale bar = 10 μm. (K) Average intensity of BiP fluorescence signal in α‐cells. Data are expressed as mean ± SEM and analyzed by one‐way ANOVA (B, F, and K), Kruskal–Wallis (C, E, G, H), and 2‐way ANOVA (D), *p < 0.05, **p < 0.01, ***p < 0.001. ER, endoplasmic reticulum; M, mitochondria; N, nucleus. Black arrows: Secretory granules; dashed line: Plasma membrane.

### 4.6 Aging Effects on α‐Cell Identity

ER stress and loss of β‐cell identity have been found to be associated in the pathophysiology of T2DM and, more recently, in age‐related β‐cell dysfunction (Talchai et al. 2012; Shrestha et al. 2022). To assess whether this association may occur in pancreatic α‐cells, we first analyzed in isolated islets the mRNA expression of key genes and transcriptional factors that regulate α‐cell identity and function such as *Arx*, *MafB*, and *Sclc2a1* (Glut1). No significant changes were observed in bulk islet mRNA levels between controls and aged groups (Figure 6A). Likewise, re‐analysis of the aforementioned scRNAseq datasets (Hrovatin et al. 2023) showed similar levels of *Arx* and *Pdx1* expression in α‐cells across age groups, despite a subtle reduction in *Gcg* and a minor increase in *MafB* expression (Figure S5), indicating that transcriptomic identity is relatively well preserved at the single‐cell level. Given the key role of Arx as a master regulator of α‐cells (Gromada et al. 2018), we also examined its presence in Gcg^+^ cells by immunofluorescence (Figure 6B). A modest decrease in the proportion of Gcg^+^ cells co‐stained for Arx was found in the islets from aged‐HIS mice and a similar trend in the aged‐LIS group (Figure 6C). We also analyzed the expression of Pdx1, which showed a major localization in Gcg^−^ cells and a negligible presence in Gcg^+^ cells (Figure S6). Finally, to further assess potential alterations in cell identity, the presence of double‐positive Ins^+^Gcg^+^ cells was evaluated in the pancreatic sections. As shown in Figure S7, aged‐LIS mice showed a higher proportion of Ins^+^Gcg^+^ cells. Taken together, these findings suggest that aging induces moderate alterations in α‐cell identity, particularly in the context of IR.

**FIGURE 6:**
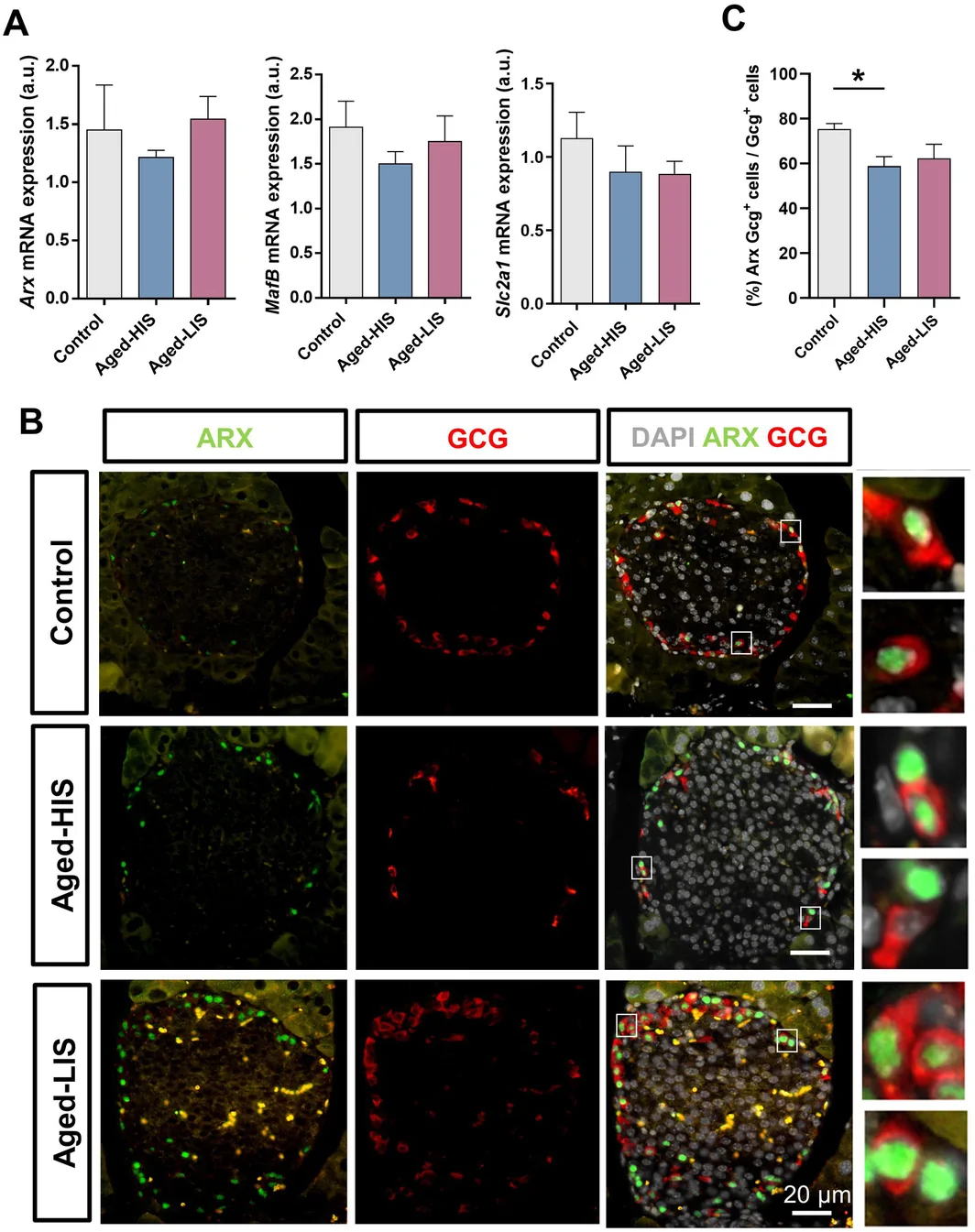
Effects of aging on α‐cell identity. mRNA levels analyzed by rtPCR in isolated islets showing the expression of the α‐cell transcription factors and identity genes *Arx*, *MafB*, and *Sclc2a1* (Glut1) (A). Representative immunofluorescence images of islets in pancreatic sections from the three groups showing Arx (green), glucagon (red) and DAPI staining (gray) (B). In the aged‐LIS images, erythrocytes show broad‐spectrum autofluorescence across all channels, creating an apparent yellow signal in merged images (Whittington and Wray 2017). They were easily distinguished from true ARX staining, restricted to the green channel, and excluded from quantitative analyses. Proportion of Arx^+^ Gcg^+^ cells respect to total Gcg^+^ cells (C). Gcg^+^ cells were analyzed according to the presence or absence of Arx staining in the nuclei identified with DAPI. Scale bar = 20 μm. Data are expressed as mean ± SEM (*n* = 62 control islets; 77 aged‐HIS islets and 62 aged‐LIS islets from 5 mice/group). One‐way ANOVA, *p < 0.05.

### 4.7 Hyperglucagonemia Is Present in Older Adults With IR and Increases the Risk to Develop T2DM in the CORDIOPREV Population

To determine whether hyperglucagonemia and altered α‐cell secretion were also present in humans with aging, we analyzed previously collected datasets from the CORDIOPREV study on plasma glucagon levels and metabolic parameters associated with glucose homeostasis. These clinical data were obtained as we previously reported (Roncero‐Ramos et al. 2018; Pozuelo‐Sanchez et al. 2020). Given the population characteristics and statistical power considerations, we first classified individuals as adults or older adults based on telomere length, a surrogate for biological age (Table 1) (Ojeda‐Rodriguez et al. 2023, 2024). Subsequently, they were divided into those who developed T2DM at the end of the study and those who did not. Individuals with T2DM incidence were characterized by IR, as shown by the HOMA‐IR index. Remarkably, plasma glucagon levels as well as the glucagon secretory response (AUC Glucagon) during an oral glucose tolerance test were higher in older adults with IR compared with the rest of the groups. We obtained similar results when classifying individuals by chronological age (Table 2).

**TABLE 1:** Plasma glucagon and related metabolic parameters categorized by biological age (telomere length *p* < 20 cutoff) and T2D incidence in the CORDIOPREV study.

	Adults (*n* = 278)	Adults T2D‐incident (*n* = 77)	Older adults (*n* = 60)	Older adults T2D‐incident (*n* = 28)	*p*
Fasting glucose (mg/dL)	92.32 ± 9.90^a^	95.69 ± 10.71	93.03 ± 9.27	98.03 ± 10.72^b^	**0.005**
Fasting insulin (mU/l)	8.28 ± 5.87^a^	10.34 ± 7.31	8.77 ± 5.36	11.24 ± 5.30^b^	**0.010**
Fasting glucagon (pg/mL)	107.54 ± 131.10^a^	124.44 ± 112.80^a^	102.69 ± 101.02^a^	235.64 ± 266.91^b^	**< 0.001**
Ratio glucagon/glucose	1.17 ± 1.40^a^	1.35 ± 1.19^a^	1.10 ± 1.10^a^	2.33 ± 2.57^b^	**0.001**
Ratio glucagon/Insulin	34.83 ± 114.14	25.50 ± 48.04	21.51 ± 33.82	32.34 ± 39.59	0.727
AUC Glucagon	13338.94 ± 13355.72^a^	16240.75 ± 14779.57^a^	12165.18 ± 11733.84^a^	25471.31 ± 21750.68^b^	**< 0.001**
HOMA‐IR	1.9 ± 1.43^a^	2.46 ± 1.85^b^	2.05 ± 1.31	2.70 ± 1.32^b^	**0.005**

*Note:* Variables are represented as means ± SD. Variables were compared using the analysis ANCOVA, adjusted for gender, body weight, statin treatment, and family history of diabetes. Different letters indicate significant differences (*p* < 0.05) between groups in the post hoc Bonferroni's multiple comparison tests. Bold indicates *p* < 0.05.

Abbreviations: AUC, area under curve; HOMA‐IR, homeostatic model assessment insulin resistance.

**TABLE 2:** Plasma glucagon and related metabolic parameters categorized by chronological age (*p* < 25 cutoff, 51 years) and T2D incidence in the CORDIOPREV study.

	Adults (*n* = 103)	Adults T2D‐Incident (*n* = 27)	Older adults (*n* = 252)	Older adults, T2D‐incident (*n* = 80)	*p*
Fasting glucose (mg/dL)	92.13 ± 9.17^a^	91.38 ± 11.2^a^	92.78 ± 10.31^a^	97.76 ± 9.98^b^	**< 0.001**
Fasting insulin (mU/l)	8.44 ± 5.58	10.94 ± 7.93	8.30 ± 5.81^a^	10.36 ± 6.40^b^	**0.048**
Fasting glucagon (pg/mL)	100.13 ± 115.75^a^	130.69 ± 99.94	106.80 ± 127.29^a^	161.46 ± 190.17^b^	**0.005**
Ratio glucagon/glucose	1.10 ± 1.25^a^	1.52 ± 1.15	1.16 ± 1.36^a^	1.64 ± 1.86^b^	**0.019**
Ratio glucagon/Insulin	42.29 ± 129.78	33.20 ± 49.11	27.55 ± 88.94	25.23 ± 44.48	0.352
AUC Glucagon	11939.17 ± 11172.23^a^	14631.69 ± 9889.09	14011.39 ± 15696.29^a^	19957.08 ± 18818.57^b^	**0.002**
HOMA‐IR	1.95 ± 1.34	2.52 ± 2.01	1.93 ± 1.43^a^	2.50 ± 1.62^b^	**0.008**

*Note:* Variables are represented as means ± SD. Variables were compared using the analysis ANCOVA, adjusted for gender, body weight, statin treatment, and family history of diabetes. Different letters indicate significant differences (*p* < 0.05) between groups in the post hoc Bonferroni's multiple comparison tests. Bold indicates *p* < 0.05.

Abbreviations: AUC, area under curve; HOMA‐IR, homeostatic model assessment insulin resistance.

Finally, we evaluated the probability of T2DM development using the Kaplan–Meier failure estimates. Individuals were classified into two age groups (adults and older adults according to the median chronological age, 58 years) and further subdivided according to their glucagon levels (low and high, according to the median value, 68.6 pg/mL). Taken together, the results suggest that age alone already confers a significant increase in the risk of developing T2DM, and that glucagon excess further amplifies this risk (Figure S8).

## 5 Discussion

Glucose intolerance and T2DM arise from reduced peripheral insulin sensitivity and insufficient compensatory response by β‐cells, processes that naturally deteriorate with age, leading to impaired glucose homeostasis (Chia et al. 2018; Tudurí, Soriano, Almagro, Montanya, et al. 2022). Most studies on age‐related diabetes have focused on β‐cells and insulin actions. However, the impact of aging on α‐cells and glucagon remains unknown. These endocrine cells play a key role in the control of glucose levels through the hepatic actions of glucagon and additionally regulate other important functions such as paracrine stimulation of β‐cells, hepatic fat metabolism, and amino acid levels, among others (Gromada et al. 2018; Capozzi et al. 2022). Furthermore, it is widely accepted that α‐cell dysfunction contributes to the dysregulation of glucose homeostasis and T2DM (Gilon 2020; Gromada et al. 2018; Capozzi et al. 2022). Patients with T2DM often present with elevated plasma glucagon levels (absolute or relative to insulin) and impaired suppression of glucagon secretion in response to glucose (Gilon 2020; Gromada et al. 2018). These alterations promote excessive glucagon signaling and hepatic glucose production, contributing to hyperglycemia. Indeed, the limitation of hepatic glucagon signaling in diabetes has generated considerable interest as a therapeutic strategy (Capozzi et al. 2022). However, although aging is a major risk factor for the development of T2DM, its repercussion on α‐cells is poorly understood. In this study, we show that aging affects the function of these endocrine cells and the regulation of glucagon.

Islet functional responses are influenced by peripheral insulin sensitivity, which can vary with age (Chia et al. 2018). To distinguish between effects produced by aging itself and those that might be due to compensatory mechanisms for IR, we analyzed aged animals with insulin sensitivity similar to that of young controls (aged‐HIS) and those with lower insulin sensitivity (aged‐LIS), as previously described (Tudurí, Soriano, Almagro, Garcia‐Heredia, et al. 2022). Remarkably, both aged groups developed hyperglucagonemia and an increased in vivo glucagon secretory response, regardless of IR. These findings are consistent with a few studies that indirectly observed high basal glucagon levels in aged mice (Talchai et al. 2012; Fan et al. 2011). Although our in vivo experiments indicate that aging itself has an impact, the ex vivo results from the present study show that IR in aged mice exacerbates age‐related alterations in α‐cells.

The increased glucagon levels in aged mice can be explained in part by the increased α‐cell mass observed in these groups, a finding consistent with a previous publication (Talchai et al. 2012). In contrast, α‐cell mass in humans remains relatively unchanged with age (Henquin and Rahier 2011). Proliferation in α‐cells was markedly reduced in old animals, a general feature of aging observed in numerous cell types, including α and β‐cells in humans and mice (Lam et al. 2018; Tudurí, Soriano, Almagro, Montanya, et al. 2022). As previously reported in aged β‐cells (Tudurí, Soriano, Almagro, Garcia‐Heredia, et al. 2022), α‐cell apoptosis occurred at very low rates, with a slight but statistically significant increase with age. Given that α‐cells are long‐lived cells with low turnover (Cnop et al. 2010), the increase in α‐cell mass likely reflects the accumulation of these cells formed during earlier life stages, which persists despite the subsequent decline in proliferation and the low‐grade apoptosis, as has been previously suggested for β‐cells (Tudurí, Soriano, Almagro, Montanya, et al. 2022). Thus, these findings may represent a snapshot of an advanced, steady‐state phase of aging, rather than the kinetics that led to expanded α‐cell mass. A higher islet proportion of α‐to‐β‐cells and α‐cells not located at the islet periphery was also evident in aged‐LIS mice. Similar changes have been frequently reported in T2DM and in conditions of metabolic stress (Masini et al. 2017; Merino et al. 2022). Given that islet cell type composition and architecture are key to the paracrine regulation of α‐cells (Gilon 2020; Gromada et al. 2018), these morphological changes could be contributing to the deficient inhibitory pattern observed in the ex vivo glucagon secretion from aged‐LIS islets.

Under physiological conditions, both insulin and high glucose concentrations repress α‐cell activity (Gilon 2020; Gromada et al. 2018). However, these regulatory cues are impaired in T2DM, resulting in reduced suppression of glucagon release, contributing to postprandial hyperglycemia (Gilon 2020; Omar‐Hmeadi et al. 2020). A similar situation was found in islets of aged‐LIS mice, where both insulin and glucose failed to produce a significant inhibition compared with controls. These results suggest that old α‐cells exhibit impaired glucose sensing/metabolism and/or defective paracrine signaling (Omar‐Hmeadi et al. 2020), which manifests primarily under IR. Collectively, this secretory profile together with the data of voltage‐dependent currents, capacitance, and plasma glucagon point to impaired inhibitory control, rather than a generalized secretory defect, as one of the main causes of α‐cell deregulation. While ex vivo secretion experiments primarily capture intrinsic and paracrine α‐cell signaling, plasma glucagon dynamics rely on broader systemic regulation, including counterregulatory hormones, neural inputs, and systemic factors associated with aging, such as inflammation (Brawerman et al. 2022; Capozzi et al. 2022; Gilon 2020; Gromada et al. 2018). Since the present study focused primarily on α‐cell alterations, future work should determine how the functional response integrates all these levels of regulation in aging. Islets from aged animals also showed a higher protein content of glucagon (particularly, the aged‐HIS group), likely as an adaptive response to the increased synthesis requirements due to the elevated glucagon release in these animals (Yau et al. 2021). This finding is consistent with a previous study documenting increased glucagon content in the whole pancreas of aged mice (Talchai et al. 2012). Since no changes in preproglucagon expression were observed, this adaptation in glucagon content may be the result of post‐transcriptional regulation (Wicksteed et al. 2003).

Ultrastructural analysis of α‐cells revealed no significant differences in the number, density, and composition of secretory granules, denoting that maturation and biogenesis of glucagon vesicles were mostly unaffected by age (Yau et al. 2021). The modest increase in granule diameter and reduced number of vesicles in contact with the plasma membrane in aged‐LIS α‐cells could reflect primary signs of cell exhaustion following compensatory hypersecretion (Yau et al. 2021). The most striking ultrastructural feature was that α‐cells from both aged groups had an enlarged ER, a hallmark of ER stress (Marroqui et al. 2015; Eizirik et al. 2020; Masini et al. 2017). Indeed, we also observed increased BiP expression in aged α‐cells. Our analysis of scRNAseq data from public databases further substantiated this, as key ER stress genes were upregulated in α‐cells. Importantly, recent studies show that both the ER organelle and the ER stress response play unique roles in several aspects of α‐cell regulation and may differ from those described in β‐cells (Maestas et al. 2024; Tsuno et al. 2025). For instance, changes in the α‐cell expression of the ER protein XBP1 induce ER stress without apparent effects on α‐cell survival (Akiyama et al. 2013). Likewise, increased α‐cell expression of the ER‐related proapoptotic protein CHOP is not necessarily coupled to α‐cell apoptosis (Tsuno et al. 2025). Furthermore, induction of ER stress in stem‐cell‐derived α‐cells increases glucagon release rather than blunting its secretion (Shrestha et al. 2025). In line with these studies, despite the enlarged ER in α‐cells from both aged groups, we did not detect a remarkable change in their apoptosis, which remained at very low rates (≈0.1%–0.2%). Additionally, in contrast to β‐cells that exhibit compromised function under chronic ER stress (Marroqui et al. 2015; Eizirik et al. 2020), the α‐cell secretory capacity at stimulatory conditions (0.5 mM glucose) was generally unaffected in aged islets. Overall, these findings are more compatible with an adaptive rather than proapoptotic ER stress state.

Similarly to T2DM, aging has also been associated with decreased expression of transcription factors that control β‐cell identity and maturity (Talchai et al. 2012; Avrahami et al. 2015; Tudurí, Soriano, Almagro, Montanya, et al. 2022), accompanied by ER stress (Shrestha et al. 2022; Brusco et al. 2023). In the case of α‐cells, however, no changes in islet expression of *Arx*, *MafB*, and *Slc2a1* were observed in aged mice. Likewise, the results obtained from the scRNAseq analysis of *Arx* and *Pdx1* from the public dataset (Hrovatin et al. 2023), as well as the immunofluorescence examination of Pdx1 in pancreas slices revealed no differences. Nevertheless, the moderate decrease in the proportion of Arx^+^Gcg^+^ cells in old mice suggests that α‐cell identity is not totally immune to aging. In any case, this does not appear to restrain the α‐cell secretory capacity. Indeed, given that the expression of *MafB*, a regulator of glucagon production and secretion, was found to be slightly upregulated in the scRNAseq data, there may be compensatory processes that result in relatively well‐preserved α‐cell identity and secretion in aging, especially compared to β‐cells (Tudurí, Soriano, Almagro, Montanya, et al. 2022). Interestingly, aged‐LIS mice displayed an increased frequency of Ins^+^Gcg^+^ cells, aligning with previous observations in insulin‐resistant humans (Mezza et al. 2016), elderly rhesus monkeys (Zhu et al. 2023), and old mice (Dubey et al. 2026). Thus, while core α‐cell genes remained relatively stable at the bulk transcriptomic level, the higher prevalence of bihormonal cells also suggests that α‐cell identity may be affected in our aged mice, particularly under IR conditions. Indeed, loss of β‐cell identity is increasingly recognized as a hallmark of islet dysfunction under chronic metabolic stress (Talchai et al. 2012; Kang et al. 2025), IR (Mezza et al. 2016), or chronological aging (Talchai et al. 2012; Shrestha et al. 2022; Iwasaki et al. 2026). Nevertheless, the precise origin and functional significance of this bihormonal cells in our model remains to be determined. Lineage‐tracing studies will be essential to clarify whether they arise from dedifferentiation, cell reprogramming or alternative processes (Son and Accili 2023).

In pancreatic β‐cells, ER stress and aging have been linked to identity loss and impaired insulin biosynthesis and secretion (Groen et al. 2021; Chen et al. 2022; Shrestha et al. 2022; Brusco et al. 2023; Maestas et al. 2024). In α‐cells, although a direct causal link remains unproven, emerging evidence suggests that ER stress affects α‐cell identity and secretory responses (Shrestha et al. 2025; Tsuno et al. 2025). Furthermore, ER stress has been shown to enhance glucagon secretion in stem‐cell‐derived α‐cells (Shrestha et al. 2025). Therefore, it is plausible that ER stress might contribute to altered glucagon regulation and the α‐cell phenotype in aged mice. Whether ER stress, α‐cell identity, and deregulated glucagon are causally connected or represent parallel events remains a possibility that warrants further investigation.

To find out whether hyperglucagonemia also occurs in humans with aging, we analyzed glucagon and glucose homeostasis data from the CORDIOPREV clinical trial (Ojeda‐Rodriguez et al. 2023, 2024). We have previously reported that α‐cell dysfunction is associated with the development of prediabetes and T2DM in this study population, and confers a higher risk of disease onset (Roncero‐Ramos et al. 2018; Pozuelo‐Sanchez et al. 2020). Using a similar approach, we observed in the present study that older adults with IR had higher basal plasma glucagon levels and increased AUC glucagon during an oral glucose tolerance test, revealing α‐cell dysfunction in this group. Notably, similar results were observed when the population was categorized either by biological or chronological age (Ojeda‐Rodriguez et al. 2023, 2024). Finally, our analysis estimated that, while age already confers a significant risk of developing T2DM, elevated glucagon levels amplify this risk. Overall, our findings would support the implication of α‐cell dysfunction in age‐associated diabetes. Additionally, these observations indicate that α‐cell alterations in human aging would manifest under additional stress conditions such as IR (Kawamori 2017). It would be important to analyze whether these changes are also observed in other study populations.

Pancreatic α‐cells exhibit innate resilience to metabolic, immune, and diabetogenic stress, in contrast to the greater vulnerability of β‐cells in diabetes (Marroqui et al. 2015). In aging, although some interspecies differences exist between humans and mice, important changes in β‐cell identity and function have been linked to impaired β‐cell secretion and/or adaptive responses (Tudurí, Soriano, Almagro, Montanya, et al. 2022; Avrahami et al. 2015; Shrestha et al. 2022). In this context, our findings suggest that α‐cells present greater resilience to aging than β‐cells, exhibiting moderate changes in identity and no decline in secretory capacity. Indeed, aging promoted higher glucagon levels, which should be detrimental to the regulation of glucose homeostasis, particularly in cases of IR or deteriorated insulin secretion, conditions frequently found in older people.

The observed phenotype in α‐cells may stem from intrinsic effects of aging, secondary systemic metabolic alterations, or a combination of both. These direct and indirect drivers may include: (1) ER stress and/or changes in α‐cell metabolism may act as an autonomous cellular trigger associated with altered secretory function and identity (Shrestha et al. 2025; Tsuno et al. 2025); (2) disrupted α‐cell spatial architecture, abnormal β and δ‐cell secretion, or aberrant expression of insulin and somatostatin receptors could alter paracrine α‐cell regulation (Kawamori et al. 2009; Kawamori 2017; Gromada et al. 2018; Gilon 2020; Omar‐Hmeadi et al. 2020); (3) senescence‐associated secretory phenotype (SASP) factors released by neighboring senescent β‐cells could affect α‐cell function (Brawerman et al. 2022; Iwasaki et al. 2026); (4) systemic metabolic and hormonal shifts resulting from aging, low‐grade inflammation, or IR may play an indirect role in the impaired α‐cell responses (Mezza et al. 2016; Staels et al. 2024). Because most of the observed effects exacerbated in aged‐LIS mice, IR appears to be a major driver of α‐cell remodeling. However, several changes were also observed in aged‐HIS animals, suggesting that aging itself is also a contributing factor. While this study has explored some of these aspects, further research is needed to fully understand these plausible direct and secondary effects and how they are integrated in α‐cell aging.

This study has some limitations, primarily related to our experimental model. Maintaining mice up to 20 months of age results in low yields. Because aged‐HIS mice represent a smaller proportion of the aged cohort, the number of available animals for this specific group was particularly constrained. Additionally, α‐cells constitute a minor fraction of total mouse islet cells, which inherently limits their isolation, purification and yield for molecular analyses. Although further mechanistic studies are required, defining α‐cell responses in aging under both insulin‐sensitive and insulin‐resistant conditions remains critical. The present comprehensive dataset offers a solid foundation for future targeted interventions, such as inducible and cell‐specific genetic models to further dissect intrinsic chronological aging from secondary systemic metabolic signals in α‐cells. Another limitation of our study is the lack of detailed electrophysiological characterization, such as direct measurements of K_ATP_ currents and other channels involved in glucagon secretion. This is particularly relevant given that altered α‐cell function may stem from impaired glucose metabolism. Therefore, future functional profiling of specific ion channel currents will be essential to fully elucidate the ionic mechanisms underlying α‐cell dysfunction in aging.

In summary, the present findings indicate that aging impacts α‐cell biology and glucagon, and that these endocrine cells are overlooked players contributing to the dysregulation of glucose metabolism with advancing age. Given glucagon's role in other metabolic functions beyond glucose homeostasis (Capozzi et al. 2022), or in longevity (Bruner et al. 2025), it would be interesting to explore other glucagon actions that might be affected in older adults or whether this age group could benefit from glucagon‐based strategies for diabetes treatment (Capozzi et al. 2022).

## Author Contributions

E.T., L.A., A.O.‐R., R.P.‐M., H.B., S.S., T.B.‐B., S.V.‐A., J.A.d.S.J., M.C.‐M., and B.M.: conducted research and/or analyzed data; E.T., A.R., I.C.‐C., A.N., P.A.‐M., B.M., J.L.‐M., and I.Q.: conceptualization and research design; B.M., A.R., I.C.‐C., A.N., P.A.‐M., B.M., J.L.‐M., and I.Q.: funding acquisition, project administration; E.T. and I.Q.: original draft and writing. All authors read and approved the manuscript.

## Funding

This work was supported by grants from the Agencia Estatal de Investigación (AEI), Ministerio de Economía y Competitividad and Ministerio de Ciencia, Innovación y Universidades (AGL2015‐67896‐P; PID2019‐104362RB‐I00; PID2023‐1468910B‐I00; PID2023‐146795OB‐I00; PID2020‐113112RB‐I00; PID2023‐150827OA‐I00), Generalitat Valenciana (CIPROM/2023/27), and National Council for Scientific and Technological Development (CNPq 306955/2023‐7).

## Conflicts of Interest

The authors declare no conflicts of interest.

## Supporting information


**Table S1:** Primer sequences employed for qPCR.

**Figure S1:** Ex vivo static glucagon secretion from freshly isolated islets normalized by glucagon content (*n* = 10–11 batches from 3 mice/group). Islets were stimulated with either low glucose (0.5 mM G, stimulatory), high glucose (11 mM G, inhibitory) or low glucose and insulin (10 nM, inhibitory). Data are expressed as mean ± SEM and analyzed by two‐way ANOVA. *p < 0.05, ***p < 0.001 respect to control (0.5 mM G).
**Figure S2:** Pancreatic α‐cells from aged mice display similar voltage‐gated K^+^ and Ca^2+^ currents compared with controls. (A) Representative recordings of voltage‐gated K^+^ and Ca^2+^ currents in response to 500 ms depolarizing pulses (−60 mV to +80 mV from a holding potential of −70 mV) in control, aged‐HIS, and aged‐LIS α‐cells. The expanded traces in aged‐HIS α‐cells show the outward K^+^ and inward Ca^2+^ currents induced by depolarization. (B) Relationship between peak voltage‐gated K^+^ current density (currents in pA normalized to cell size in pF) and voltage of pulses in control (*n* = 14 α‐cells from 4 mice), aged‐HIS (*n* = 14 α‐cells from 3 mice), and aged‐LIS (*n* = 10 α‐cells from 3 mice) animals. (C) Relationship between voltage‐gated Ca^2+^ current density (currents in pA normalized to cell size in pF) and voltage of pulses in control (*n* = 10 α‐cells from 4 mice), aged‐HIS (*n* = 6 α‐cells from 3 mice), and aged‐LIS (*n* = 9 α‐cells from 3 mice) animals. Data are expressed as mean ± SEM and analyzed by two‐way repeated‐measures ANOVA (B and C).
**Figure S3:** Pancreatic α‐cells from aged mice display similar voltage‐induced capacitance changes compared with controls. (A) Representative recordings of capacitance in response to a train of 20 × 500 ms depolarizing pulses from −70 to 0 mV. (B) Quantification of exocytosis (capacitance difference normalized by cell size) from 14 controls and 16 aged α‐cells from 2 mice per group. Isolated α‐cells were characterized as previously reported (Junior et al. 2025). Membrane capacitance was monitored with the standard whole‐cell configuration through the sine +DC mode of the Lock‐In amplifier included in Patch Master software in an EPC‐10 amplifier (HEKA). Currents were filtered using a 4‐pole Bessel filter and digitized at 20 kHz. The extracellular medium contained (in mM): 138 NaCl, 5.6 KCl, 2.6 CaCl_2_, 1.2 MgCl_2_, 5 HEPES, and 5 D‐glucose (pH 7.4). Electrodes were filled with internal solution contained (in mM): 125 K‐ Glutamate, 10 KCl, 10 NaCl, 1 MgCl_2_, 0.05 EGTA, 3 Mg‐ATP, 0.1 cAMP, and 5 HEPES (pH 7.1). Data are expressed as mean ± SEM. Non‐significant (ns). Note that, in this specific experiment, aged 20‐month‐old mice were not stratified according to insulin sensitivity.
**Figure S4:** (A) Illustrative immunofluorescence images of islets in pancreas slices showing Xbp1 (green), glucagon (red) and DAPI staining (gray). The dashed line on the left images indicates the magnified area shown in the images on the right. The contour of pancreatic α‐cells is indicated by a dashed line on the right images. (B) Average intensity of Xbp1 fluorescence signal in α‐cells. Images are representative of 35 islets from 3 controls, 32 islets from 3 aged‐HIS mice and 31 islets from 3 aged‐LIS mice. Data are expressed as mean ± SEM. One‐way ANOVA. Non‐significant (ns). Scale bar = 10 μm.
**Figure S5:** Single‐cell RNAseq from public dataset (Hrovatin et al. 2023) showing the expression of *Arx*, *Pdx1*, *Gcg*, and *MafB* genes in pancreatic α‐cells from young adults and old mice.
**Figure S6:** Illustrative immunofluorescence images of islets in pancreas slices from the three groups showing Pdx1 (green), glucagon (red) and DAPI staining (gray). Images are representative of 37 islets from 4 controls, 50 islets from 3 aged‐HIS and 16 islets from 2 aged‐LIS mice. Scale bar = 20 μm.
**Figure S7:** (A) Illustrative immunofluorescence images of islets in pancreas slices showing insulin (green), glucagon (red) and DAPI (blue) staining. The white squares on the islets indicates the magnified area shown in the images on the right for each animal group. The magnified images show the staining for insulin, glucagon, and the merge image. (B) Percentage of Ins^+^Gcg^+^ of total islet cells per group. Images are representative of 45–55 islets from 5 mice per animal group. Data are expressed as mean ± SEM. Statistical analysis were performed using one‐way ANOVA. **p < 0.01 respect to controls. Scale bar = 20 μm.
**Figure S8:** Risk of incident T2DM according to age and glucagon levels. Kaplan–Meier failure estimates showing the probability of T2DM development and associated hazard ratio in individuals of the CORDIOPREV study categorized in adults and older adults and distributed in low and high plasma glucagon levels (established by median values). Data are expressed as HR (95% CI) and analyzed by Cox regression adjusted for gender, body weight, statin treatment, and family history of diabetes. *p < 0.05, **p < 0.01.

## Data Availability

The data that support the findings of this study are available on request from the corresponding authors.
