# Supplementary material for: Aging Affects Pancreatic α‐Cell Function and Promotes Hyperglucagonemia: Implications in Age‐Associated Diabetes
Source: Aging Cell. 2026 Oct 6;25(10):e70753. doi: 10.1111/acel.70753 (PMC13640062; doi:10.1111/acel.70753)
Supplement: Supplementary file 1 [file ACEL-25-e70753-s002.pdf]

Supplemental Figure 1

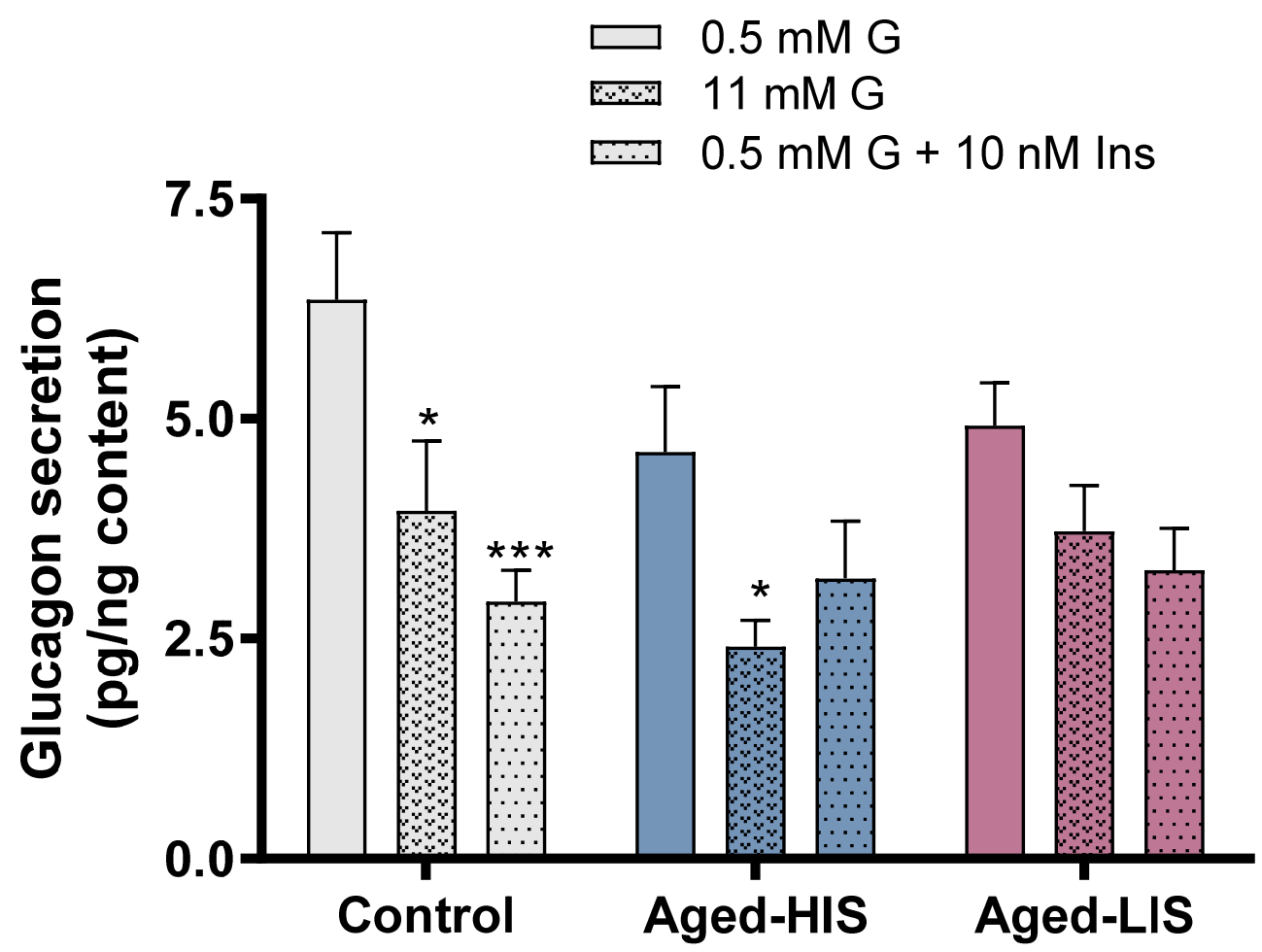

Supplemental Figure 1. *Ex vivo* static glucagon secretion from freshly isolated islets normalized by glucagon content (n = 10-11 batches from 3 mice/group). Islets were stimulated with either low glucose (0.5 mM G, stimulatory), high glucose (11 mM G, inhibitory) or low glucose and insulin (10 nM, inhibitory). Data are expressed as mean ± SEM and analyzed by two-way ANOVA. *$p < 0.05$, ***$p < 0.001$ respect to control (0.5 mM G).

Supplemental Figure 2

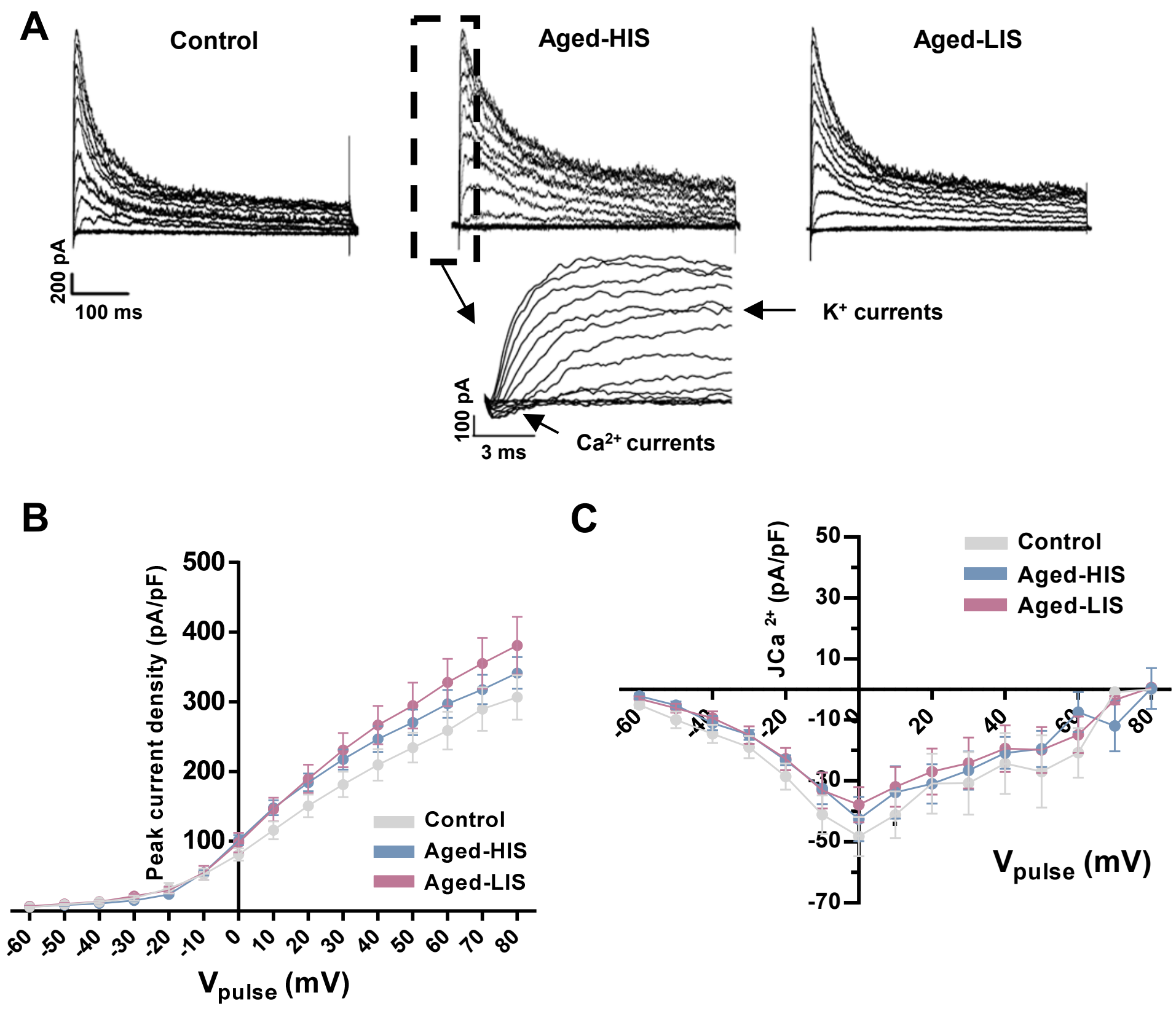

Supplemental Figure 2. Pancreatic α-cells from aged mice display similar voltage-gated K^+ and Ca^{2+} currents compared with controls. A) Representative recordings of voltage-gated K^+ and Ca^{2+} currents in response to 500 ms depolarizing pulses (−60 mV to +80 mV from a holding potential of −70 mV) in control, aged-HIS, and aged-LIS α-cells. The expanded traces in aged-HIS α-cells show the outward K^+ and inward Ca^{2+} currents induced by depolarization. **B)** Relationship between peak voltage-gated K^+ current density (currents in pA normalized to cell size in pF) and voltage of pulses in control (n=14 α-cells from 4 mice), aged-HIS (n=14 α-cells from 3 mice), and aged-LIS (n=10 α-cells from 3 mice) animals. **C)** Relationship between voltage-gated Ca^{2+} current density (currents in pA normalized to cell size in pF) and voltage of pulses in control (n=10 α-cells from 4 mice), aged-HIS (n=6 α-cells from 3 mice), and aged-LIS (n=9 α-cells from 3 mice) animals. Data are expressed as mean ± SEM and analyzed by two-way repeated-measures ANOVA (B and C).

Supplemental Figure 3

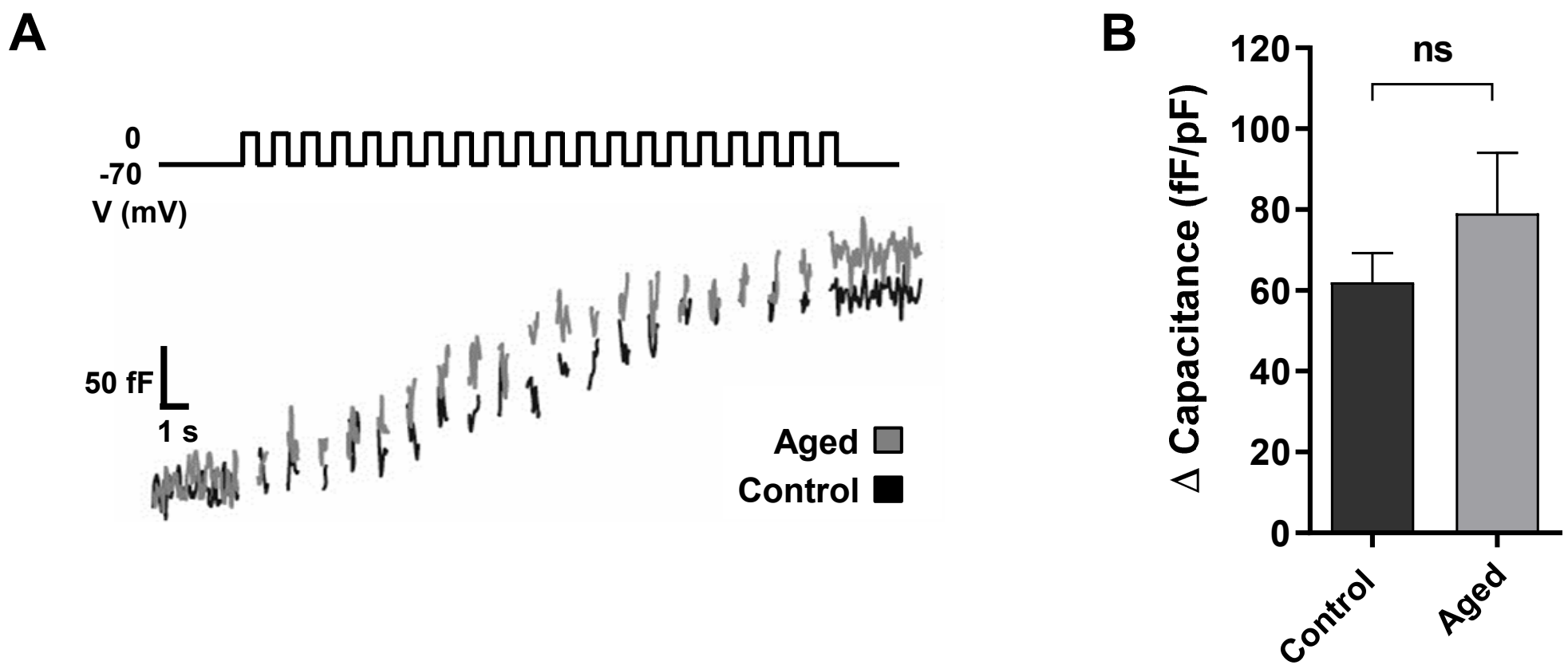

Supplemental Figure 3. Pancreatic α-cells from aged mice display similar voltage-induced capacitance changes compared with controls. A) Representative recordings of capacitance in response to a train of 20x500 ms depolarizing pulses from -70 to 0 mV. **B)** Quantification of exocytosis (capacitance difference normalized by cell size) from 14 controls and 16 aged α-cells from 2 mice per group. Isolated α-cells were characterized as previously reported (Junior da Silva JA et al. 2025). Membrane capacitance was monitored with the standard whole-cell configuration through the sine +DC mode of the Lock-In amplifier included in Patch Master software in an EPC-10 amplifier (HEKA). Currents were filtered using a 4-pole Bessel filter and digitized at 20 kHz. The extracellular medium contained (in mM): 138 NaCl, 5.6 KCl, 2.6 $CaCl_2$, 1.2 $MgCl_2$, 5 HEPES, and 5 $_D$-glucose (pH 7.4). Electrodes were filled with internal solution contained (in mM): 125 K- Glutamate, 10 KCl, 10 NaCl, 1 $MgCl_2$, 0.05 EGTA, 3 Mg-ATP, 0.1 cAMP and 5 HEPES (pH 7.1). Data are expressed as mean ± SEM. Non-significant (ns). Note that, in this specific experiment, aged 20-month-old mice were not stratified according to insulin sensitivity.

Supplemental Figure 4

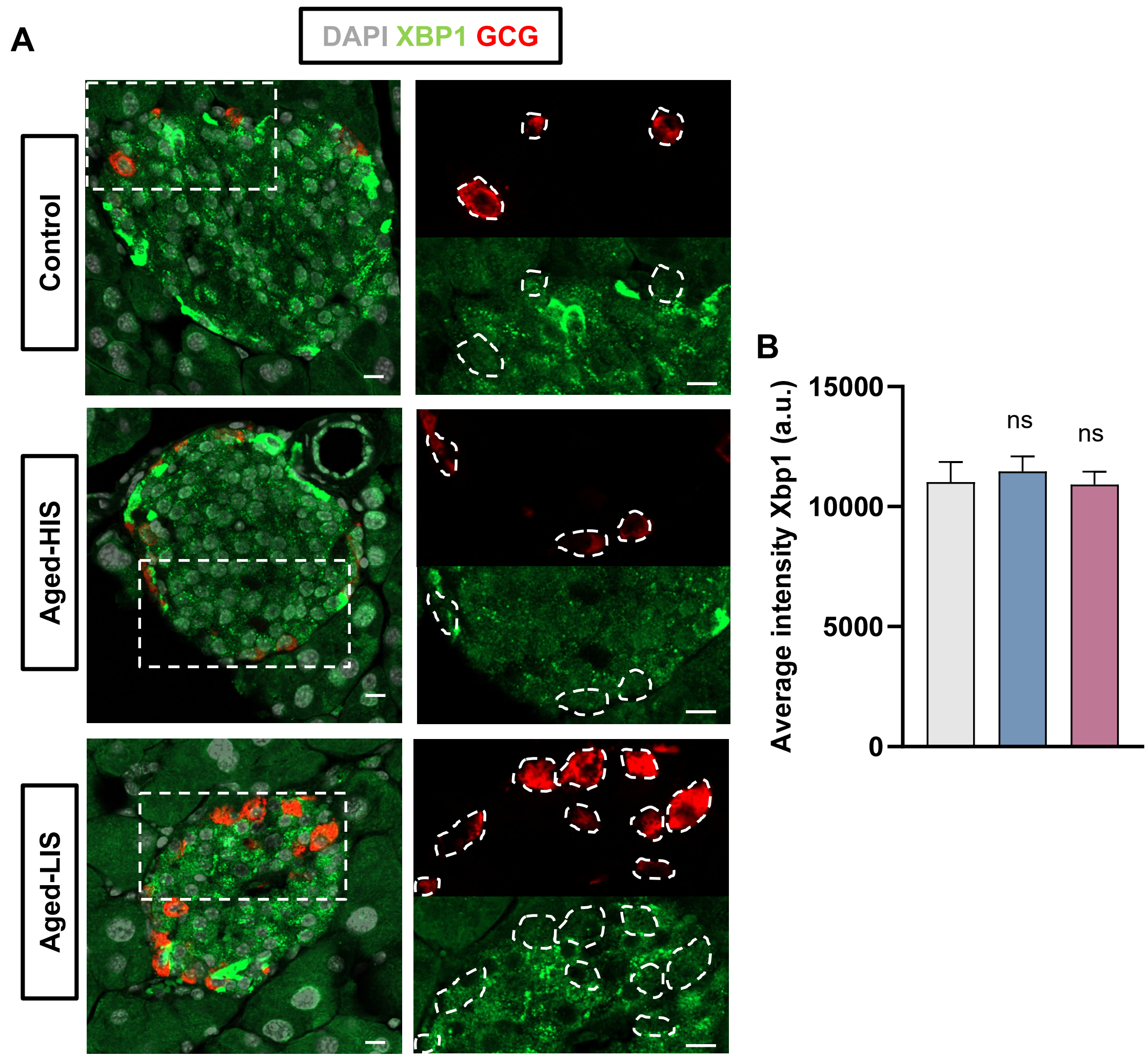

Supplemental Figure 4. **A)** Illustrative immunofluorescence images of islets in pancreas slices showing Xbp1 (green), glucagon (red) and DAPI staining (grey). The dashed line on the left images indicates the magnified area shown in the images on the right. The contour of pancreatic α-cells is indicated by a dashed line on the right images. **B)** Average intensity of Xbp1 fluorescence signal in α-cells. Images are representative of 35 islets from 3 controls, 32 islets from 3 aged-HIS mice and 31 islets from 3 aged-LIS mice. Data are expressed as mean ± SEM. One-way ANOVA. Non-significant (ns). Scale bar= 10 µm.

Supplemental Figure 5

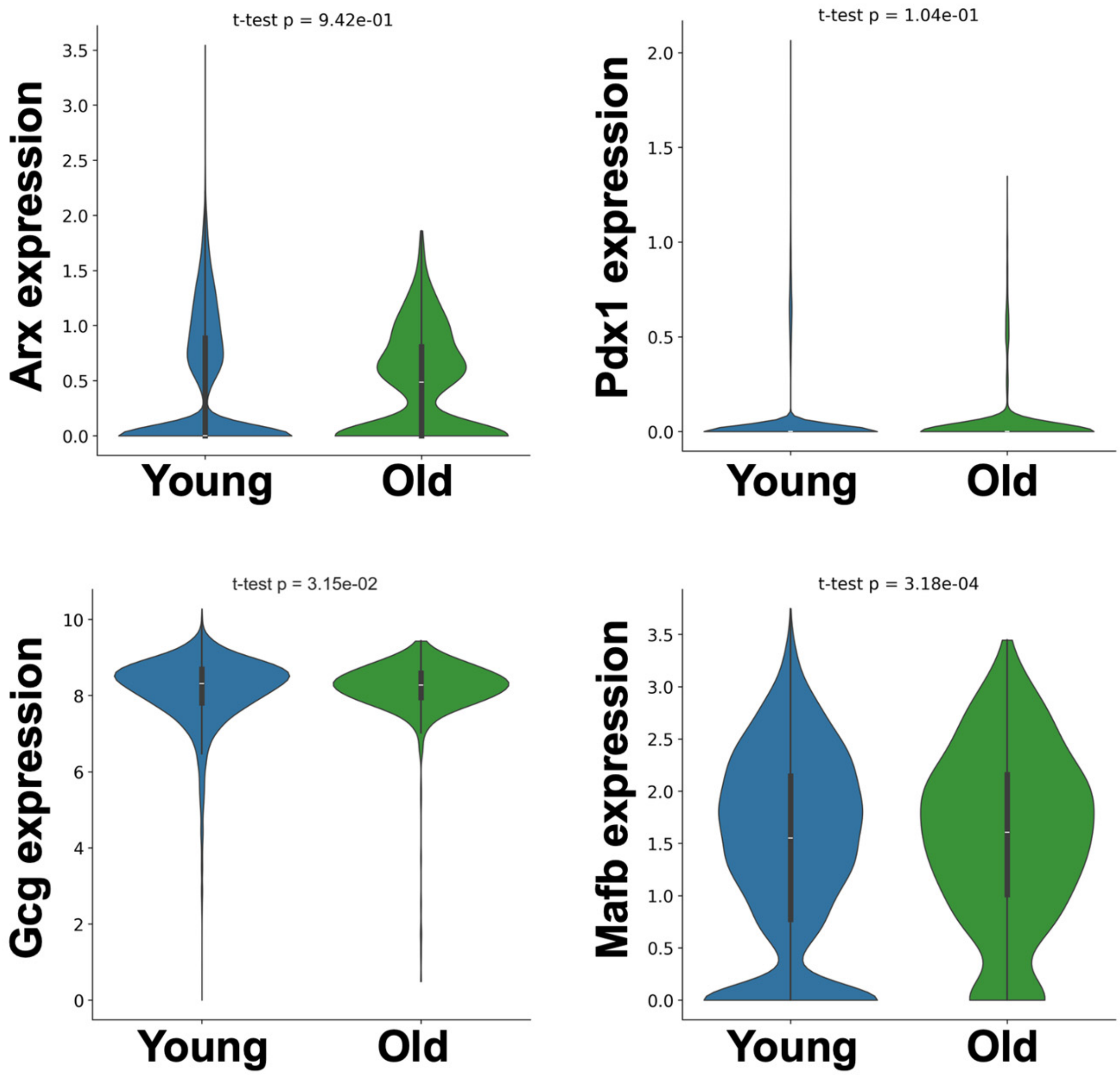

Supplemental Figure 5. Single-cell RNAseq from public dataset (Hrovatin et al. 2023) showing the expression of *Arx*, *Pdx1*, *Gcg* and *MafB* genes in pancreatic α-cells from young adults and old mice.

Supplemental Figure 6

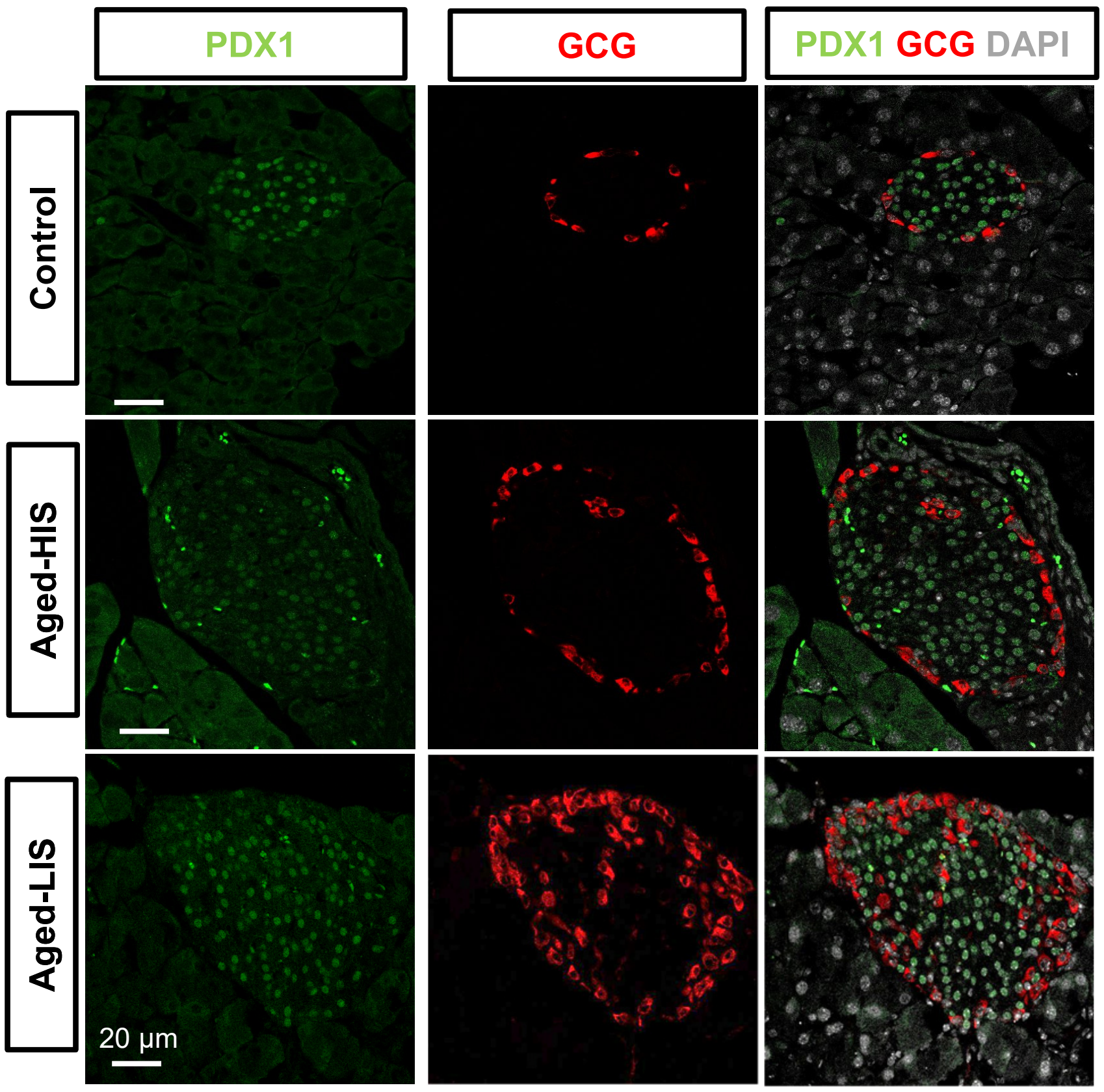

Supplemental Figure 6. Illustrative immunofluorescence images of islets in pancreas slices from the three groups showing Pdx1 (green), glucagon (red) and DAPI staining (grey). Images are representative of 37 islets from 4 controls, 50 islets from 3 aged-HIS and 16 islets from 2 aged-LIS mice. Scale bar=20 µm.

Supplemental Figure 7

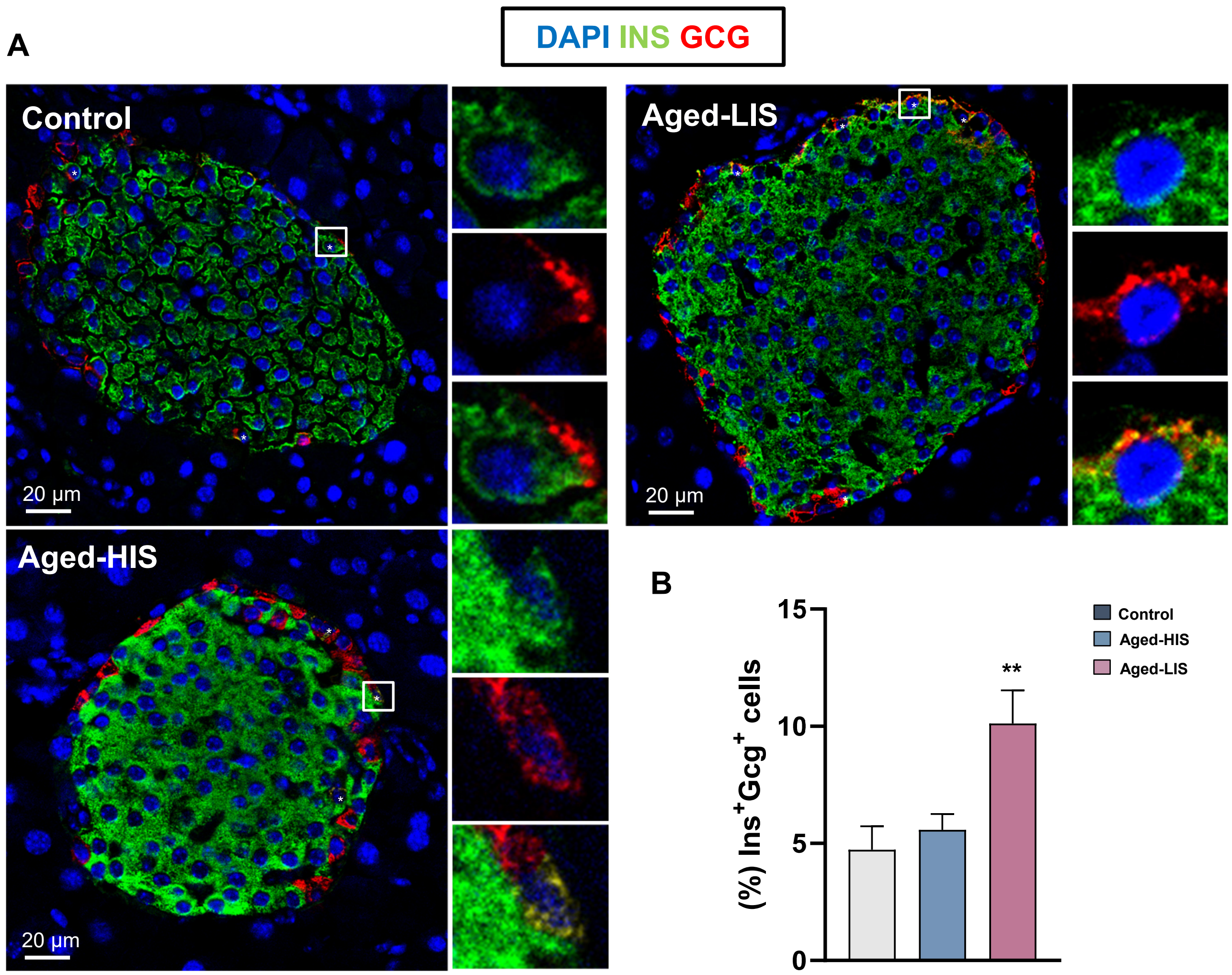

Supplemental Figure 7. **A)** Illustrative immunofluorescence images of islets in pancreas slices showing insulin (green), glucagon (red) and DAPI (blue) staining. The white squares on the islets indicates the magnified area shown in the images on the right for each animal group. The magnified images show the staining for insulin, glucagon, and the merge image. **B)** Percentage of Ins^+Gcg^+ of total islet cells per group. Images are representative of 45-55 islets from 5 mice per animal group. Data are expressed as mean ± SEM. Statistical analysis were performed using one-way ANOVA. **$p < 0.01$ respect to controls. Scale bar= 20 µm.

Supplemental Figure 8

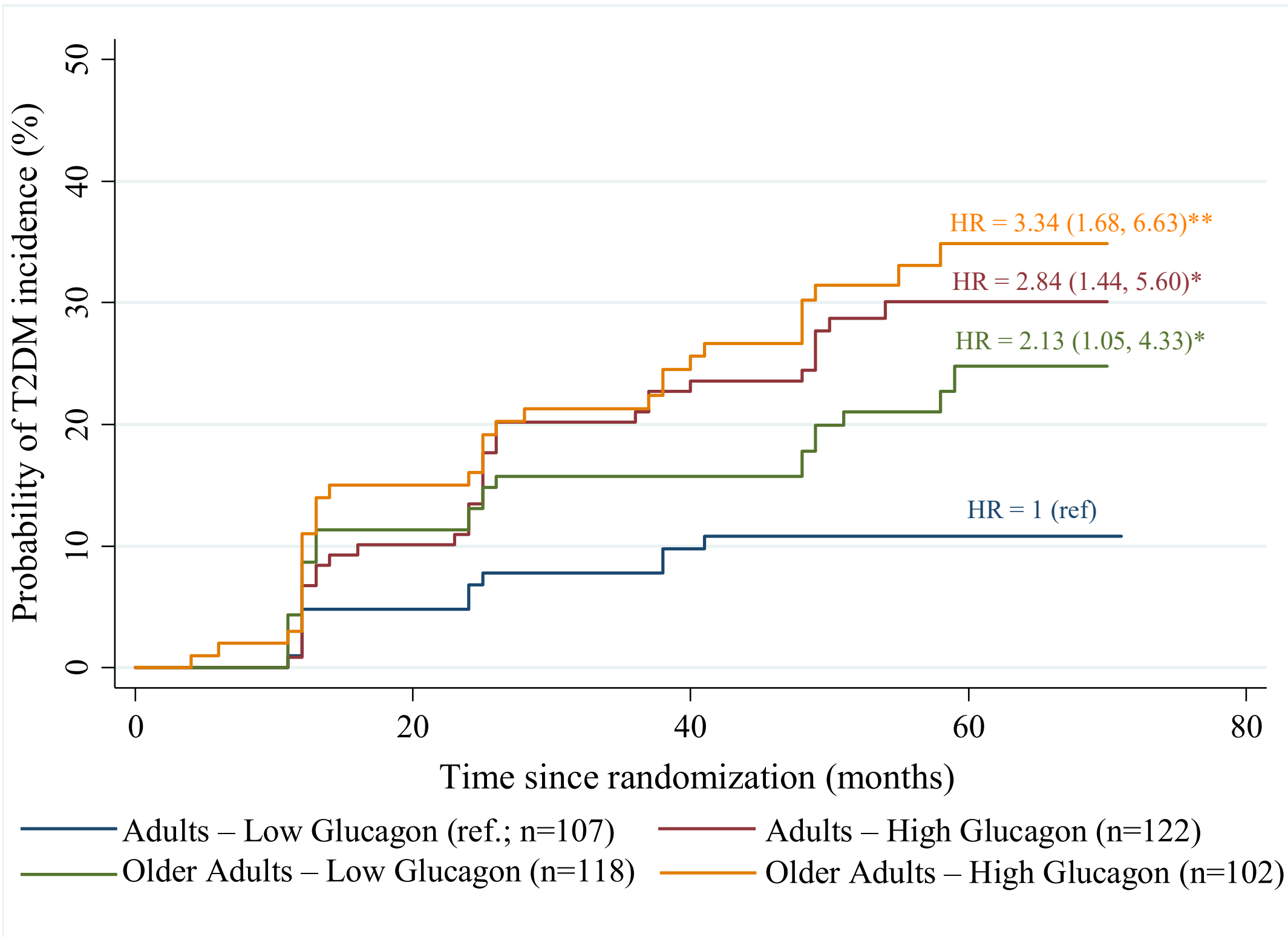

Supplemental Figure 8. **Risk of incident T2DM according to age and glucagon levels.** Kaplan-Meier failure estimates showing the probability of T2DM development and associated hazard ratio in individuals of the CORDIOPREV study categorized in adults and older adults and distributed in low and high plasma glucagon levels (established by median values). Data are expressed as HR (95% CI) and analyzed by Cox regression adjusted for gender, body weight, statin treatment, and family history of diabetes. *$p<0.05$, **$p<0.01$.
