# Supplementary material for: Aging Affects Pancreatic α‐Cell Function and Promotes Hyperglucagonemia: Implications in Age‐Associated Diabetes
Source: Aging Cell. 2026 Oct 6;25(10):e70753. doi: 10.1111/acel.70753 (PMC13640062; doi:10.1111/acel.70753)
Supplement: Supplementary file 2 [file ACEL-25-e70753-s001.docx]

**Supplemental Table 1**

Primer sequences employed for qPCR.

| Gene | Forward (5’- 3’) | Reverse (5’-3’) | REFSEQ (NM) |
| --- | --- | --- | --- |
| *Arx* | GGCCGGAGTGCAAGAGTAAAT | TGCATGGCTTTTTCCTGGTCA | 001305940.1 |
| *Gcg* | CACTCACAGGGCACATTCAC | TTTGGCAATGTTGTTCCGGTT | 008100.4 |
| *Hprt* | GGTTAAGCAGTACAGCCCCA | TCCAACACTTCGAGAGGTCC | 013556.2 |
| *MafB* | ACCAAGGACGAGGTGATCC | CAGGTGATGTTTCTGCTGGA | 010658.3 |
| *Slc2a1* | TACACCCCAGAACCAATGGC | CCCGTAGCTCAGATCGTCAC | 011400.3 |
